# The Functional Differences between the GroEL Chaperonin of *Escherichia coli* and the HtpB Chaperonin of *Legionella pneumophila* Can Be Mapped to Specific Amino Acid Residues

**DOI:** 10.3390/biom12010059

**Published:** 2021-12-31

**Authors:** Karla N. Valenzuela-Valderas, Gabriel Moreno-Hagelsieb, John R. Rohde, Rafael A. Garduño

**Affiliations:** 1 Department of Microbiology and Immunology, Dalhousie University, Sir Charles Tupper Medical Building, 7th Floor, 5850 College Street, Halifax, NS B3H 1X5, Canada; karla.valenzuela.tm@gmail.com (K.N.V.-V.); John.Rohde@dal.ca (J.R.R.); 2 Department of Biology, Room BA441, Wilfrid Laurier University, 75 University Avenue West, Waterloo, ON N2L 3C5, Canada; gmoreno@wlu.ca

**Keywords:** chaperonin, GroEL, evolutionary trace, ECM29, yeast-two-hybrid, Legionella, HtpB, site-directed mutagenesis

## Abstract

Group I chaperonins are a highly conserved family of essential proteins that self-assemble into molecular nanoboxes that mediate the folding of cytoplasmic proteins in bacteria and organelles. GroEL, the chaperonin of *Escherichia coli*, is the archetype of the family. Protein folding-independent functions have been described for numerous chaperonins, including HtpB, the chaperonin of the bacterial pathogen *Legionella pneumophila*. Several protein folding-independent functions attributed to HtpB are not shared by GroEL, suggesting that differences in the amino acid (aa) sequence between these two proteins could correlate with functional differences. GroEL and HtpB differ in 137 scattered aa positions. Using the Evolutionary Trace (ET) bioinformatics method, site-directed mutagenesis, and a functional reporter test based upon a yeast-two-hybrid interaction with the eukaryotic protein ECM29, it was determined that out of those 137 aa, ten (M68, M212, S236, K298, N507 and the cluster AEHKD in positions 471-475) were involved in the interaction of HtpB with ECM29. GroEL was completely unable to interact with ECM29, but when GroEL was modified at those 10 aa positions, to display the HtpB aa, it acquired a weak ability to interact with ECM29. This constitutes proof of concept that the unique functional abilities of HtpB can be mapped to specific aa positions.

## 1. Introduction

Group I chaperonins constitute a large family of highly conserved ~60-kDa essential proteins that typically reside in the bacterial cytoplasm, or the organellar matrix. They team-up with the equally conserved ~10-kDa essential co-chaperonins, to self-assemble into molecular nanoboxes that mediate the folding of cytoplasmic proteins, and have recently found some biotechnological applications, as reviewed by Horwich and Fenton, and Pipaón et al. [1,2]. Each nanobox comprises a barrel of 14 chaperonin subunits (in turn formed by two heptameric rings), and two lids (each formed by seven co-chaperonin subunits) that close the barrel ends. Inside the closed barrel, favorable conditions are provided for the folding of nascent polypeptides, or the re-folding of denatured proteins, in an ATP-mediated process [1,2,3,4]. The GroEL/GroES protein folding machinery of the bacterium *Escherichia coli* is the best characterized group I chaperonin/co-chaperonin complex.

In spite of their sequence and structural conservation, imposed by their essential protein folding function, bacterial 60-kDa chaperonins show a diversity of protein folding-independent functions, as reviewed by Henderson and Martin [5]. Although it is not known whether chaperonins from free-living bacteria can display protein folding-independent functions, so far such functions have only been described in chaperonins of bacterial pathogens and bacterial symbionts. For example, *Mycobacterium tuberculosis* Hsp60.2 is found exposed on the bacterial cell surface where it acts as a ligand of CD43 on macrophages [6], *Helicobacter pylori* secretes its Hsp60, which in turn has a role in iron binding [7], and the chaperonin of the insect symbiont *Buchnera aphidicola* (also known as symbionin) has kinase activity [8], and contributes to the transmission of some plant viruses [9]. Secreted and surface-exposed chaperonins of bacterial pathogens are also able to activate intra- and inter-cellular signaling pathways in eukaryotic cells [10,11], a topic also reviewed by Henderson and Martin [5].

The 60-kDa chaperonin of the intracellular bacterial pathogen *Legionella pneumophila* (known as HtpB) is a *bona fide* chaperonin with protein-folding activity [12] that assembles into double ring barrels, seen by electron microscopy [13]; being 75.5% identical to the Group I chaperonin archetype GroEL. In addition, HtpB is a secreted and a surface-associated protein [14] that plays a potential role in pathogenesis through a variety of protein folding-independent functions, as reviewed by Garduño et al., and Garduño and Chong [15,16]. Polystyrene micro-beads coated with HtpB (but not GroEL) are efficiently taken up by HeLa and CHO cells [17,18]. HtpB purified from *L. pneumophila*, and recombinant HtpB either purified from *E. coli* or expressed in mammalian cells (but not GroEL) attract mitochondria and modulate the structure of CHO cells’ actin cytoskeleton [18]. When recombinant HtpB (but not GroEL) is expressed in yeast (*Saccharomyces cerevisiae*), it induces pseudohyphal growth, indicating that HtpB directly or indirectly triggers a eukaryotic signal transduction pathway [12,19]. Finally, overexpression of HtpB (but not GroEL) in Gram-negative bacteria induces filamentation [20], as reviewed by Garduño and Chong [16]. It is thus obvious that HtpB displays a variety of protein folding-independent functions not shared by the *E. coli* GroEL.

Ever since we learned that HtpB alters eukaryotic signalling and reaches the cytoplasm of mammalian host cells [21,22], as reported by Nasrallah et al. [19], systematic yeast-two-hybrid (Y2H) screens have been conducted to identify eukaryotic cytoplasmic proteins that interact with an HtpB bait [19,21,22,23,24], reviewed by Valenzuela-Valderas et al. [12]. The experimental hypothesis behind these Y2H screens was that HtpB must interact with eukaryotic protein partners and(or) receptors, to directly or indirectly mediate the entry of *L. pneumophila* into host cells, trigger internal signalling pathways in host cells, and alter organelle trafficking. One of the proteins identified in these screens corresponded to the C-terminus of the human homolog of the proteasome adaptor and scaffold protein ECM29 (hECM29) [24]. While several of the proteins identified in those Y2H screens could also interact with GroEL, the GroEL-hECM29 interaction was negative. Therefore, the Y2H interaction of HtpB with hECM29 is of particular interest, simply because it could provide an unequivocal, practical assay to functionally distinguish HtpB and GroEL.

ECM29 is a large 200–210 kDa eukaryotic protein involved both in the assembly of the 26S proteasome and the regulation of its activity [25]. The 26S eukaryotic proteasome is a multi-protein complex formed by a 19S regulatory particle that recognises and unfolds ubiquitinated protein substrates, and a 20S core particle that degrades substrates into short peptides to be reused by the cell [26,27]. The 26S proteasome thus plays a major role in the maintenance of a healthy proteome in eukaryotic cells [28,29]. ECM29 is composed almost entirely of HEAT-like repeats [30], which function as flexible domains that can wrap around target proteins helping them to assemble [31], thereby giving ECM29 its scaffolding qualities. ECM29 has a large protein interactome [32], including 227 unique partners listed in the *Saccharomyces* Genome Database (https://www.yeastgenome.org (accessed on 24 December 2021)). Consequently, by interacting with ECM29, HtpB could potentially interface with eukaryotic cellular processes that could benefit the intracellular replication of *L. pneumophila*.

The hypothesis behind the work presented here is that the protein folding-independent functions of HtpB are the result of mutations leading to substitutions in key amino acids (aa) not present in GroEL. Since HtpB and GroEL differ in 137 scattered aa, we used the well-validated evolutionary trace (ET) method [33] to narrow down the number of HtpB aa most likely responsible for its unique functions. Forty-one functionally important aa positions were identified in HtpB, from which 10 were selected for further experimentation through mutagenesis. The Y2H interaction of HtpB with hECM29 was validated here as a robust functional reporter, to test the effect of site-directed mutations in the 10 selected aa positions. Alanine substitutions in four of the ten selected residues (K298, H473, K474 and N507) most affected the interaction of HtpB with hECM29. In addition, when all 10 selected residues were substituted in GroEL by the corresponding aa found in HtpB, GroEL became marginally proficient at interacting with hECM29. The reported results constitute proof of concept that functional abilities can be linked to specific HtpB aa positions, and also suggest that the ability of HtpB to interact with eukaryotic host cell proteins is an acquired trait evolved from substitutions in specific aa positions.

## 2. Materials and Methods

### 2.1. Microbial Strains and Growth Conditions

*E. coli* strains DH5α and JM109, described by Sambrook et al. [34] were grown at 37 °C in Lysogeny broth (LB) or on LB plates solidified with 2% agar. Transformants of DH5α carrying constructs made in pBluescript (pBS, Agilent Technologies, Inc., Santa Clara CA, USA) or pGBKT7 (Clontech, Cat. No. 630489. Clontech is now Takara Bio USA Inc.) were selected in LB with ampicillin (100 μg/mL) or kanamycin (50 μg/mL), respectively. *L. pneumophila* strain JR32 was grown at 37 °C on (N-[2-acetamido]-2-aminoethane-sulfonic acid)—buffered charcoal yeast extract (BCYE) broth [35], and only used to obtain genomic DNA. Bacterial stocks were maintained at −80 °C in Nutrient Broth containing 20% (*v*/*v*) glycerol.

The yeast strain used was *Saccharomyces cerevisiae* Y2Hgold (Clontech, Cat No. 630498, San Jose, CA, USA), which is designed for use with the Clontech’s Matchmaker™ Gold Yeast Two-Hybrid System (Clontech, Cat. No. 630489, San Jose, CA, USA). The Y2HGold strain (subsequently referred to simply as ‘yeast’) contains four distinct reporter genes (*HIS3, ADE2, MEL1* and *AUR1-C*) that are only expressed in the presence of Gal4-based protein interactions. *HIS3* is needed for the biosynthesis of histidine and is controlled by the Gal4-responsive promoter G1. *ADE2* (needed for the biosynthesis of adenine) is controlled by the G2 promoter. *MEL1* encodes α-galactosidase that turns colonies blue in growth media containing X-alpha-Gal (Clontech, Cat. No. 630463, San Jose, CA, USA), and *AUR1-C* encodes resistance to aureobasidin A. Both *AUR1-C* and *MEL1* share the same Gal4-responsive promoter M1. This strain was used to perform all the yeast-two-hybrid (Y2H) assays with the various HtpB and GroEL baits (below). Yeast was typically grown for 3 days at 30 °C in YPD agar plates [in g/L: yeast extract 10, peptone (BD, Cat. No. 211677, Mississauga, ON, Canada) 20, glucose 20, agar 15] or in synthetic defined (SD) media (Supplemental Table S1). Stock plates were kept at 4 °C for up to a month. Frozen yeast stocks were kept at −80 °C in Nutrient Broth containing 20% (*v*/*v*) glycerol, or in the appropriate SD medium containing 25% glycerol.

### 2.2. General Molecular Biology Methods

These were performed following the basic principles and instructions described by Sambrook et al. [34]. Briefly, DNA gel electrophoresis was done in 1 or 2% agarose gels in TAE buffer and stained with 1 μg/mL ethidium bromide. A VersaDoc™ MP 5000 System (Bio Rad Laboratories Inc., Mississauga, ON, Canada) was used for visualization, and gel images were saved as JPEG files. Genomic DNA was purified from bacterial cell pellets by the phenol:chloroform (1:1) method. Genomic DNA pellets were thoroughly rinsed with 70% ethanol and air-dried. Before use, dry genomic DNA was solubilized in nuclease-free deionized water (Invitrogen, Cat. No. 10977, Ottawa, ON, Canada. Invitrogen is now part of Thermo Fisher Scientific). Plasmid and PCR amplicon purification was carried out using commercial kits from Qiagen Inc. (Cat. No. 27104 and Cat. No. 28704, respectively, Toronto, ON, Canada) according to the manufacturer’s instructions. All restriction endonucleases were from New England BioLabs Ltd. (NEB Canada, Whitby, ON, Canada), and restriction reactions were done according to NEB instructions. Restricted DNA fragments were separated by electrophoresis and purified directly from agarose gels using the Qiagen (Cat. No. 28704, Toronto, ON, Canada) gel purification kit. Ligation reactions were carried overnight using NEB T4 DNA ligase (Cat. No. M0202, Whitby, ON, Canada) following manufacturer’s indications. Transformation of electrocompetent DH5α cells was done by electroporation with 2 mm gap cuvettes in a MicroPulser™ apparatus (Bio-Rad Laboratories, Mississauga, ON, Canada) at 2.5 kV for 5 milliseconds. After electroporation, cells were incubated at 37 °C for 1 h with shaking and then plated onto pre-warmed (37 °C) LB selective plates.

### 2.3. Polymerase Chain Reactions (PCR)

These were routinely done in a Biometra GmbH T1 thermocycler (sourced from Montreal Biotech Inc., Kirkland, PQ, Canada), using NEB Taq DNA Polymerase (Cat. No. M0267, Whitby, ON, Canada), or Platinum^®^ Pfx DNA Polymerase (Invitrogen, Cat. No. 11708, Ottawa, ON, Canada) following manufacturer’s indications. All PCR primers were from Integrated DNA Technologies, Inc., and their sequences are specified in Table 1. PCR primers solubilized in nuclease-free water were used at a final concentration of 10 μM. A typical 25 μL PCR reaction consisted of 18.4 μL nuclease-free water, 2.5 μL 10X ThermoPol Buffer, 1 μL dNTP mix (NEB, Cat. No. N0446S, Whitby, ON, Canada), 1 μL of the 10 μM solution of each primer, 1 μL template DNA and 0.125 μL *Taq* DNA Polymerase. For 50 μL PCR reactions the aforementioned amounts were doubled. Thermo-cycling conditions were as follows: initial denaturation at 94 °C for 5 min, 30 cycles of [94 °C for 15 s, 55 to 62 °C (depending on primers Tm) for 30 s, and 68°C for 1 min per kb], and a final extension at 68 °C for 10 min. Purified amplification products (amplicons) were stored at −20 °C until use. For direct colony PCR, single bacterial colonies from agar plates were suspended in 50 μL of sterile ddH_2_O in 0.2 mL tubes (Eppendorf Ltd., Mississauga, ON, Canada), heated at 95°C for 5 min in the Biometra T1 thermocycler, and centrifuged at 16,000× *g* (MIKRO 20, Hettich, sourced from Montreal Biotech Inc. Kirkland, PQ, Canada) for 1 min. Template DNA for PCR reactions was 1 μL supernatant from the heat-lysed cells.

### 2.4. Yeast Molecular Biology Methods

For transformation, yeast grown to exponential phase (OD_600_ 0.4–0.6) in 300 mL of fresh YPD were pelleted (1000× *g* for 5 min at room temperature), washed with ddH_2_0, and resuspended in 1.5 mL of a sterile lithium acetate solution [0.1 M lithium acetate (Sigma-Aldrich Canada Co. Cat No. L-6883, Oakville, ON, Canada) in TE buffer (10 mM Tris-HCl, 1 mM EDTA, pH 7.5)] to produce competent cells. Competent yeast cells were used immediately after preparation, mixing 100 μL of cell suspension with 0.1 μg of plasmid DNA (0.1 μg of each plasmid for co-transformations) and 0.1 mg of carrier DNA (Clontech, Cat No. 630440, San Jose, CA, USA). Then, 0.6 mL of sterile PEG/LiAc solution [40% polyethylene glycol 3350 (Sigma Cat No. P-3640, Oakville ON, Canada) and 0.1 M lithium acetate in TE buffer] was added, mixed vigorously, and incubated overnight at room temperature. Next day, 70 μL of dimethyl sulfoxide (DMSO, Sigma, Cat. No. 472301, Oakville ON, Canada) was added and mixed gently by inversion. Yeast cells where then heat-shocked for 30 min at 42 °C in the Biometra T1 thermocycler, chilled 2 min on ice, and pelleted at 16,000× *g* for 5 s. Pellets of transformed yeast were resuspended in 200 μL of sterile TE buffer and plated on the appropriate selective agar plates, which were incubated for three days at 30 °C and then stored at 4 °C for up to one month. Transformed yeast cells were typically grown in SD medium, lacking specific nutrients and(or) with added selective components (Table S1).

To purify plasmids from yeast cells we used the QIAprep Spin Miniprep Kit (Qiagen, Cat No. 27104, Toronto, ON, Canada) with a mechanical disruption step according to the manufacturer’s instructions. Briefly, a 2-mL culture in the appropriate SD medium grown overnight at 30 °C was vigorously mixed in a 2.5-mL conical screw cap tube (BIOPLAS Inc., Cat. No. 4216R, sourced from VWR Canada, Mississauga, ON, Canada) with 200 μL of 0.5 mm glass beads (BioSpec, Cat. No. 11079105, sourced from Fisher Scientific, Ottawa, ON, Canada), and centrifuged at 1000× *g* for 2 min. The supernatant was then discarded, and the pellet resuspended in 250 μL of buffer P1 from the kit. Then, 250 μL of buffer P2 was added and the mixture was beaten in a Mini-BeadBeater^TM^ (sourced from Montreal Biotech Inc. Kirkland, PQ, Canada) at 4800 oscillations/min for 3 min at 4 °C (alternating 1-min beating bursts with 5-min chilling on ice), after which, 350 μL of buffer N3 was added, and the debris pelleted at 16,000× *g* for 10 min. The supernatant containing released soluble DNA was applied to a QIAprep spin column, and purified plasmid DNA was eluted in 30 μL of nuclease-free water.

### 2.5. Yeast Protein Techniques

Yeast cells from 50-mL cultures grown overnight were chilled on ice, pelleted (1000× *g*, 5 min, 4 °C), and washed once by centrifugation with ice-cold ddH_2_O. Cells were then resuspended in 1 mL of ice-cold lysis buffer (25 mM Tris-HCl, 15 mM EGTA, 1 mM EDTA, 150 mM NaCl, 0.1% Triton X-100, 10% glycerol, 1 mM DTT, 1 mM PMSF, pH 7.5), transferred to a 2.5-mL BIO PLAS conical screw cap tube containing 400 μL of 0.5 mm glass beads, and beaten for 3 min at 4 °C, using a Mini-Bead Beater^TM^ (sourced from Montreal Biotech Inc. Kirkland, PQ, Canada) at 4800 oscillations/min (alternating 1-min beating bursts with 5-min chilling on ice). Beaten cells were pelleted at 16,000× *g* for 5 min. Supernatants containing soluble proteins were quantified using the BCA Protein Assay Kit (Thermo scientific, Cat. No. 23225, sourced from Fisher Scientific, Ottawa, ON, Canada), and then stored at −80 °C until use.

Co-immunoprecipitation was done with protein extracts from yeast co-transformed with pGBK:*htpB* and pGAD:*ECM29*, which provide HtpB with a c-Myc epitope tag and ECM29 with an HA tag, respectively. In 1.5-mL microcentrifuge tubes, protein extract aliquots (corresponding to 1 mg of total protein) were incubated for 2 h at 4 °C with 1 µg of anti-HA polyclonal antibody (Clontech, Cat. No. 631207, San Jose CA, USA) or 1 µg of anti cMyc monoclonal antibody (Clontech, Cat. No. 631206, San Jose, CA, USA). Then 30 µL of protein A/G Sepharose^®^ (abcam Canada, Cat. No. ab193262, Toronto, ON, Canada) were added and the tubes were rotated gently for 2 h at 4 °C. The Sepharose beads were recovered by low-speed centrifugation (500× *g*) and washed 3× with lysis buffer (the first wash supernatant was saved). SDS-PAGE vertical slabs and subsequent immunoblotting were run in a Mini-PROTEAN^®^ apparatus (Bio-Rad Laboratories, Mississauga, ON, Canada), according to standard electrophoresis methodology. Briefly, 12 μL protein samples mixed with 3 μL of 5× sample buffer, or 7 μL of a protein ladder (NEB, Cat. No. P7712S, Whitby, ON, Canada) were run per lane in 10-well gels. After electrophoresis, gels were equilibrated in transfer buffer (3 g/L Tris base, 14.4 g/L glycine, 20% methanol) and proteins electro-transferred to an activated PVDF membrane (EMD Millipore, Cat. No. IPVH00010, Oakville ON, Canada. EMD Millipore is now Millipore/Sigma) for 90 min at 90 V in the Mini-PROTEAN^®^ apparatus. Membranes were then stained for 5 min with Ponceau S (Allied Chemical Canada Ltd., Cat. No.628, Mississauga, ON, Canada) to visually ensure proteins were transferred correctly. Membranes were then destained in PBS, blocked for 3h with 2% skim milk and 0.2% BSA in TTBS [0.5% (*v*/*v*) Tween 20, 6 g/L Tris base, 1.86 g/L EDTA, 8.77 g/L NaCl, pH 7.3), and incubated overnight at 4 °C with the primary antibody [either monoclonal anti-c-Myc (Clontech, Cat. No. 631206, San Jose, CA, USA) or polyclonal anti-HA-Tag (Clontech, Cat. No. 631207, San Jose, CA, USA)] diluted 1:500 in TTBS containing 0.01% BSA. Next day, membranes were incubated for 1 h with the corresponding alkaline phosphatase-conjugated secondary antibody [either goat anti-mouse IgG (Cedarlane, Cat.No. CLCC30008, Burlington ON, Canada) or goat anti-rabbit IgG (Cedarlane, Cat. No. CLCC42008, Burlington ON, Canada)]. Color was developed with 5-Bromo-4-chloro-3-indolyl phosphate disodium salt (BCIP, Sigma, Cat.No.B1026) and Nitro-blue tetrazolium chloride (NBT, Sigma, Cat. No. N6876, Oakville ON, Canada). Dried stained membranes were imaged in the VersaDoc™ MP 5000 System.

### 2.6. Prediction of the 3-D Protein Structure of HtpB

Since there is no experimental 3-D structure available for HtpB, the ModWeb server (https://modbase.compbio.ucsf.edu/modweb/ (last accessed on 19 December 2021)) was used to predict it. The HtpB protein sequence (GI: 52840925) was submitted and the slow-fold assignment method (Seq-Prf, PSI-Blast) was selected to calculate the sequence-structure alignment. Three models were selected by ModWeb, based on 109 detected hits, and 77 calculated models of the best hits found in the protein data bank (PDB). The 3 models selected were: the (GroEL-K-Mg-ATP)14 complex of *E. coli* (PDB code 1KP8), the Cpn60/Cpn10/(ADP)7 complex of *Thermus thermophilus* (PDB code 4V4O), and the apical domain of the GroEL-1 of *Mycobacterium tuberculosis* (PDB code 3M6C). Although the hit to the chaperonin of *Thermus thermophilus* was to a crystal structure of the entire Cpn60 (PDB code 4V4O), only 144 aa positions of the apical domain (PDB code 1SRV) showed high identity (65%) to HtpB, whereas the rest of the protein structure had a lower similarity. That is, the models based on the chaperonins of *Thermus thermophilus* and *Mycobacterium tuberculosis* did not enable reliable modelling of the full molecule. Therefore, the only structural model that included a template region encompassing 523 aa positions (nearly the full protein) with 76% identity to HtpB, was the one based on the crystal structure of the *E. coli* GroEL chaperonin, which was the model that we used for mapping amino acid positions and ET ranks. The use of the model based on the 3-D structure of GroEL was experimentally ideal, and all the more convenient, because GroEL has been consistently used as a functional reference for HtpB in all our previous studies. 

Analysis of the secondary protein structure of the HtpB aa cluster in positions 471-475, was performed using the online software VADAR (Volume, Area, Dihedral Angle Reporter) following the developers’ instructions [36].

### 2.7. Evolutionary Trace Analysis

To identify key amino acids likely implicated in the protein folding-independent functions of HtpB, we performed an evolutionary trace (ET) analysis following the protocol developed by Lichtarge and collaborators, as reported by Wilkins et al. [37]. First, to identify orthologs of HtpB (GI: 52840925), a search was performed against the National Center for Biotechnology Information (NCBI) non-redundant protein sequence database, using the Basic Local Alignment Search Tool for proteins (blastp) under default parameters. Orthologs with an E-value less than 1 × 10^−6^ were retrieved and then aligned using Clustal Ω [38]. Identical sequences were eliminated using the Usearch’s derep_fulllength command [39]. Mitochondrial Hsp60s, Archaea group II chaperonins, and sequences with less than 50% the length of HtpB were manually eliminated from the multiple sequence alignment (MSA). The HtpB MSA was then used as input for the ET code analysis, at http://evolution.lichtargelab.org/ (last accessed on 24 December 2021). Briefly, in the “Universal Evolutionary Trace” online tool, “Sequence and UniProt Accession Number” was chosen as input method, and the HtpB UniProt accession number (Q5ZXP3) was specified. Then, under “Advanced Options”, the “Real-Valued Trace (rvET)” option was selected, and the HtpB MSA in GCG format was uploaded under “Custom Sequence Input”. The ET tool builds a pairwise sequence similarity matrix using the sequences in the MSA, and then the UPGMA method is applied [40] to generate an evolutionary tree where the sequences are separated in groups according to the tree branching. A consensus sequence is established for each group/branch and then, the aa trace ranks are assigned based on the minimum number of branches into which the evolutionary tree must be partitioned for that residue to be invariant within each branch/group [37]. Finally, the invariance within the individual branches is also introduced to the calculation, to obtain the real-value ET (rvET). The rvET ranks (hereafter designated simply as ET ranks) were then mapped onto the predicted HtpB 3D structure, using PyMol and PyETV plugin (http://mammoth.bcm.tmc.edu/traceview/HelpDocs/PyETVHelp/pyInstructions.html (last accessed on 24 December 2021)). The ET rank is a relative ranking of evolutionary importance for each aa position in the MSA. Low ET ranks (the lowest value being 1.0) indicate sequence conservation, and therefore, an implied functional importance. To narrow down the search for aa residues that are more likely involved in functional divergence, we took advantage of the knowledge that most of the protein folding-independent functions of HtpB cannot be performed by GroEL. We used the BLOcks SUbstitution Matrix (BLOSUM 62) as a secondary tool to provide a relative measure of biological probability for each aa substitution between HtpB and GroEL [41]. The BLOSUM 62 score follows a numerical scale between −3 and +9. Positive scores mean conservative (more likely) substitutions, and negative scores indicate non-conservative (less likely) substitutions. Hence, the criteria to finally select HtpB aa involved in functional diversity were a combination of a low ET rank and a negative BLOSUM 62 score.

### 2.8. Cloning and Mutagenesis of htpB and groEL (Baits for the Y2H Assays)

The entire *htpB* gene (1659 bp) was PCR amplified in a 50 μL reaction using JR32 genomic DNA as template and primers *Eco*RI-htpB_F/BamHI-htpB_R. Similarly, *groEL* (1653 bp) from JM109 genomic DNA was PCR amplified with primers EcoRI-groEL_F/SalI-groEL_R. The cleaned PCR products, and plasmid pBS, were both digested with *Eco*RI and *Bam*HI for *htpB*, or *Eco*RI and *Sal*I for *groEL*, ligated to produce pBS:*htpB* and pBS:*groEL* (respectively), and transformed into *E. coli* DH5α. Once positive transformants were confirmed by colony PCR with primers EcoRI-htpB_F/BamHI-htpB_R or EcoRI-groEL_F/SalI-GroEL_R, pBS:*htpB* and pBS:*groEL* were isolated and restricted with *Eco*RI/*Bam*HI or *Eco*RI/*Sal*I, respectively. The dropped *htpB* and *groEL* fragments were then purified and ligated into *Eco*RI/*Bam*HI- or *Eco*RI/*Sal*I-restricted pGBKT7 (Clontech, Cat. No. 630489), to generate pGBK:*htpB* or pGBK:*groEL*, which were then transformed into DH5α. pGBK:*htpB* and pGBK:*groEL* constituted the basic molecular baits for the Y2H assays (below), as they carry *htpB* and *groEL* in translational frame with the *GAL4* DNA-binding domain and the c-Myc epitope tag. Both basic baits were purified from DH5α and verified by bi-directional DNA sequencing with primer sets EcoRI-htpB_F/BamHI-htpB_R, HtpB419_F/HtpB1200_R, or EcoRI-groEL_F/SalI-groEL_R, GroEL461_F/GroEL1154_R (respectively), as well as unidirectional sequencing with the T7 primer. DNA sequencing was performed by Genome Quebec (which also provided the T7 primer).

Site-directed and multisite-directed mutations in *htpB* and *groEL* were first made in pBS:*htpB* or pBS:*groEL*, as these high-copy plasmids were small (~4.5 kb) and readily available in large amounts from cultures of DH5α. Mutated genes were then subcloned into pGBKT7 (as described above for *htpB* and groEL). Single aa replacements were done with the PCR-based QuikChange^®^ Site-Directed Mutagenesis Kit (Agilent Technologies Canada Inc., Cat. No. 200518, Mississauga, ON, Canada) following the exact instructions of the manufacturer. Complementary primers targeted to introduce the desired mutations were designed using the QuikChange^®^ Primer Design Program (http://www.stratagene.com/qcprimerdesign (accessed on August 2015)). Similarly, multiple site mutations were done with the QuikChange^®^ Multi Site-Directed Mutagenesis Kit (Agilent Technologies Canada Inc., Cat. No. 200515, Mississauga, ON, Canada). For nucleotide changes that could not be covered by a single primer, multiple primers were designed (QuikChange^®^ Primer Design Program, as above) to bind the same strand of the template DNA in one PCR reaction, so that the template (parental DNA strand) could be later digested with DpnI endonuclease (NEB Cat. No. R0176, Whitby, ON, Canada), before transformation into *E. coli*. All base changes were verified by bi-directional sequencing using primer pairs: MMBD_F/MMBD_R, EcoRI-htpB_F/BamHI-htpB_R, HtpB419_F/HtpB1200_R, EcoRI-groEL_F/SalI-groEL_R, GroEL461_F/GroEL1154_R, as well as unidirectional sequencing using the T7 primer (the latter being provided by Genome Quebec, Montreal, PQ, Canada).

### 2.9. Yeast-Two-Hybrid (Y2H) Assays

The interaction of parental (wild-type, WT) or mutant HtpB and GroEL baits with hECM29 was evaluated through Y2H plate assays (where CFU spotting and colony color were determined), or through Y2H broth assays (where the optical density of broth cultures, and their corresponding α-galactosidase activity, were quantified). Yeast cells were co-transformed with one of the bait plasmids constructed in the pGBKT7 vector (above), and with the pGAD:*hECM29* plasmid, rescued from a confirmed positive clone previously identified in a Y2H screening using HtpB as bait against the Mate & Plate™ Library–Universal Human (Clontech, Cat. No. 630481, San Jose, CA, USA) [21]. The pGAD:*hECM29* plasmid contains a cDNA sequence encoding a protein fragment that maps to the C-terminus half of hECM29, that should be in translational frame with the activation domain of the yeast transcription factor Gal4.

Yeast co-transformants carrying both a bait plasmid and pGAD:*hECM29* were selected on double drop-out (DDO) SD (–Leu/–Trp) agar plates (Table S1). A single colony of each co-transformant was subsequently transferred to 2 mL of DDO SD liquid medium and incubated overnight at 30 °C. For Y2H plate assays, serial dilutions (1:10, 1:100 and 1:1000) of the 2-mL overnight cultures were then spotted (10 μL per dilution) on selective quadruple drop-out (QDO: –Ade/–His/–Leu/–Trp) SD agar plates containing 40 μg/mL X-α-Gal (Clontech, Cat. No. 630463) and 200 ng/mL aureobasidin A (Clontech, Cat. No. 630466) (QDO/X/A solid medium, Table S1). Protein interactions were qualitatively evaluated as positive (+++), impaired (++), weakly positive (+) or negative (–), based on the CFU density per spot, and the color of the colonies (blue or white). For Y2H broth assays, the 2-mL overnight culture in DDO SD was used to inoculate three 2-mL QDO SD broth cultures to achieve a starting OD_600_ of 0.8 units. The OD_600_ of these QDO cultures was measured again after 6 days of incubation at 30 °C. In addition, α-galactosidase activity was quantified using the colorimetric assay recommended in the Yeast Protocols Handbook (Clontech, PT3024-1). Briefly, the 6-day old broth cultures in QDO were diluted 1:10 with ddH_2_O and their OD_600_ recorded. Then, 1 mL of each diluted culture was centrifuged at 16,000× *g* for 1 min, and 16 μL (0.016 mL) of supernatant were loaded, in triplicate, into wells of a 96-well plate containing 48 μL of assay buffer [33 mM p-nitrophenyl α-d-galactopyranoside (PNP-α-Gal, Sigma Cat No. N0877, Oakville ON, Canada) in 0.33 M sodium acetate (Sigma Cat No. S7545), pH 4.5]. The α-galactosidase-mediated hydrolysis of the chromogenic substrate PNP-α-Gal was allowed to proceed at 30 °C for 180 min in microplates covered with a parafilm-sealed lid and stopped by adding 136 μL of 1 M sodium carbonate (the final reaction volume being 0.2 mL). Absorbance was then measured at a wavelength of 410 nm in a Benchmark Plus microplate reader. Alpha-galactosidase activity was calculated as: Milli-units per (mL × cell) = A_410_ × 0.2 × 1000/[(20.3 × 0.52) × 180 × 0.016 × 10 OD_600_], where A_410_ is the measured absorbance of each well, and OD_600_ is the optical density of each 6-day old QDO/A culture diluted 1:10.

### 2.10. Statistical Analysis

Unpaired two-tailed Student t-tests were performed to determine the statistical significance (*p*-value) of differences in OD_600_ or α-galactosidase activity (in relation to the activity of the Y2H interaction between WT HtpB and hECM29), using the R software version 4.1.1.

## 3. Results

### 3.1. Evolutionary Trace Analysis Identified 10 Amino Acids Potentially Involved in the Protein Folding-Independent Functions of HtpB

**Figure 1:**
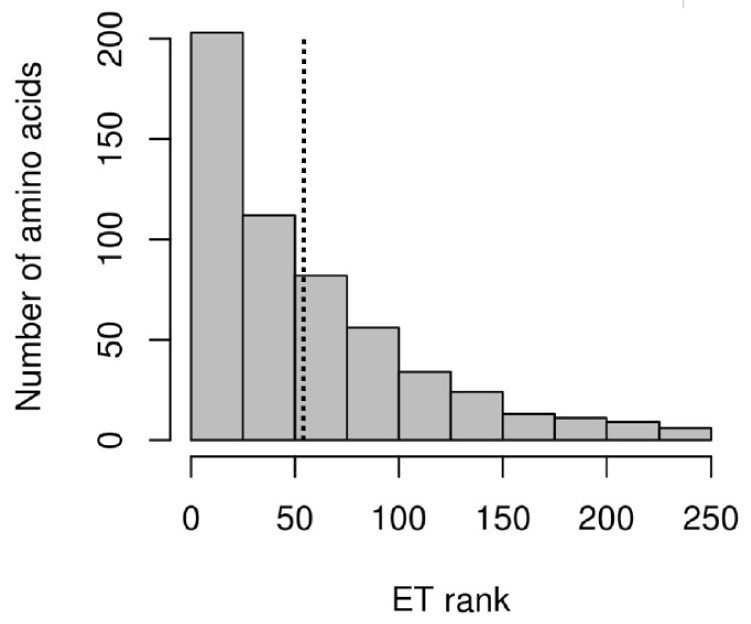
Histogram showing the distribution of ET rank values for the 550 amino acid positions of HtpB, in intervals of 25 arbitrary units (horizontal axis). The number of amino acids in each interval is represented by the bars’ heights, against the scale of the vertical axis. The mean value of the ET ranks (55.80) is marked by the dotted vertical line.

A total of 1373 sequences of bacterial Group I chaperonins were used to construct a Multiple Sequence Analysis (MSA) matrix that constituted the Evolutionary Trace (ET) analysis input. The analysis output displayed the amino acid (aa) variability per position in the complete alignment, and the ET rank for all 550 aa positions of HtpB (Table S2, Supplementary Materials). The ET ranks ranged from 1.00 (lowest possible value) to 234.92, with a median value of 41.03, and an average value of 55.80. It should be remembered that a low ET rank means that the aa present in a given position is highly conserved, i.e., an aa position with an ET rank of 1.0, means that the same aa is found in that position in all the 1373 chaperonin sequences analyzed. In fact, 27 aa positions (5% of the total positions in the MSA) had an ET rank of 1.00, and 336 (61%) of the aa positions had ranks under the average (Figure 1), with 164 positions at the top 30th percentile, clearly confirming that bacterial Group I chaperonins are highly conserved.

**Figure 2:**
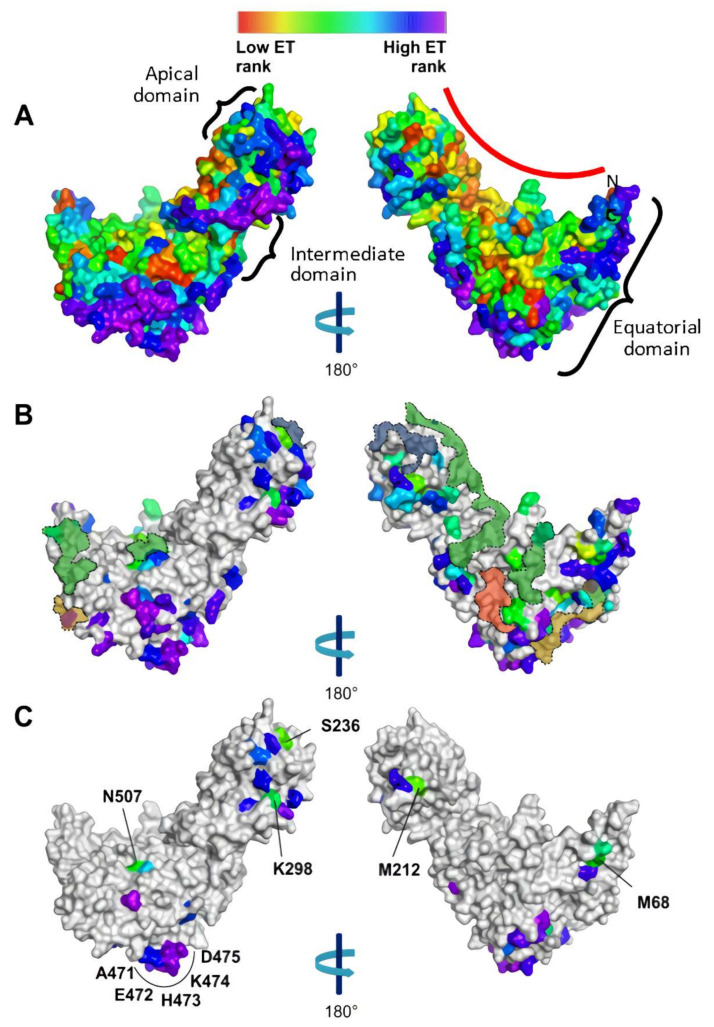
Surface representations of the 3-D predicted structure of HtpB, showing 180° rotation images created in the ModWeb server. The scale at the top shows the color code of ET ranks, from the lowest value (1 = deep red) to the highest (234.92 = bright purple) and applies to the three panels. (**A**) ET ranks of all 550 amino acids (aa) in HtpB. The three structural domains of chaperonins are indicated by braces, and the red arc shows the HtpB concave face. N and C indicate the N-terminus (first aa) and C-terminus (last aa), respectively. (**B**) ET ranks of the 137 aa positions where HtpB and GroEL differ. The locations of aa important for protein folding functions have been manually delineated with dotted outlines as follows: Blue shadow = Substrate polypeptide recognition, Red shadow = ATP-binding pocket, Green shadow = Intra-ring contacts, and Yellow shadow = Inter-ring contacts. (**C**) ET ranks of the 41 less-likely substitutions between HtpB and GroEL. Aa positions selected for functional testing are specifically labeled and correspond to the less likely substitutions either with a low (green) ET rank or present in a cluster.

A graphic visualization of the ET ranks of Table S2 is shown in Figure 2A, where the ET ranks were mapped on the predicted 3-D structural model of HtpB. It should be noted here that on this graphical representation, only those aa localized on the outer surfaces of the protein can be seen. The prevalent colors in Figure 2A are cyan, green, yellow, orange and red (corresponding to intermediate/low ET rank values), particularly in the concave surface delineated by the red arc, which in the 7-mer chaperonin ring defines the internal wall of the protein folding chamber. Conversely, the almost exclusive clustering of light blue, blue and purple on the outer (exposed) surfaces of HtpB should be noted, as these represent aa positions with a high variability (i.e., intermediate/high ET ranks) that signify low evolutionary conservation.

It seemed reasonable to hypothesize that aa conservation is imposed by the essential protein folding function of Group I chaperonins, and that aa important for this function would have a low ET rank. To test this, GroEL aa that had been already identified to have important roles in protein folding were searched for in the literature [3,42,43,44,45,46,47,48,49,50,51,52]. We found 39 GroEL aa involved in intra-ring interactions, 23 involved in ATP binding, 16 in polypeptide recognition, and 14 in inter-ring interactions. Equivalent positions of these GroEL aa were identified for HtpB, and their ET ranks tabulated (Table 2). As expected, the ET ranks for these protein folding-related aa were intermediate/low (mean value = 31.86, SD = 33.40, *n* = 92). The aa positions with the lowest ET rank values were those involved in ATP-binding (mea*n* = 12.70, SD = 20.10, *n* = 23). Among these 23 aa positions, there were seven with an ET rank value of 1.00. On the other hand, the highest ET ranks among the protein folding-related aa (i.e., the more variable positions) were those involved in inter-ring contacts (mean value = 65.05, standard. Deviatio*n* = 43.13, *n* = 14), and this group contained the aa position with the highest ET rank (146.41) of the protein folding-related aa. This variability could explain why HtpB and GroEL (among other chaperonins) are not functionally interchangeable [53,54,55]. The areas where the protein folding-related aa reside are delineated in Figure 2B. Table 2 also shows the 11 aa substitutions found between HtpB and GroEL, and it is noteworthy that even in the highly conserved ATP-binding pocket, there was one discrepancy in position 32. These 11 aa substitutions associated with the protein folding function of HtpB were thus assumed not to have a role in the functional diversity of HtpB.

Having mapped conserved aa involved in protein folding, we then solely focused on the HtpB and GroEL alignment, to attempt the identification of aa potentially involved in the protein folding-independent functions of HtpB not shared by GroEL. HtpB and GroEL are 75.5% identical, meaning that only 137 aa are different between them. The ET ranks of these 137 substitutions are mapped in Figure 2B. From these 137 aa substitutions, we selected 41 based on their negative BLOSUM 62 score (Table 3), which were then mapped on the 3-D structure of HtpB (Figure 2C). It should be remembered that a negative.

**Figure 3:**
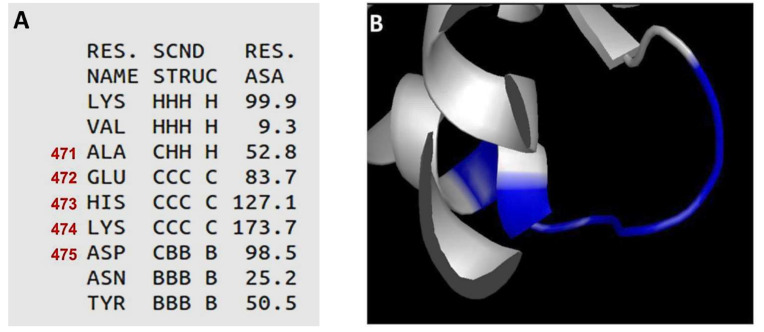
Analysis of the secondary structure of the HtpB amino acid (aa) cluster in positions 471-475. Secondary structure was predicted using VADAR and visualized using PyMol. (**A**) Table of parameters associated with the secondary structure of the HtpB polypeptide between aa positions 469-477. The positions corresponding to the AEHKD cluster are highlighted in red. Residue names (RES. NAME) are shown as three-letter codes. The secondary structure (SCND STRUC) of which each aa is part of, is denoted as follows: H = alpha helix, C = random coil and B=beta sheet; the first three letters representing the predictions by three independent methods, and the fourth letter (separated by a space) being the consensus secondary structure. The residue accessible surface area (RES. ASA) for each aa is given in square angstroms. The higher the ASA value, the more accessible the corresponding aa would be to interact with a water molecule. (**B**) Diagrammatic close-up of the ribbon structure of HtpB, showing the predicted conformation of the random coil formed by the amino acids in positions 471–475 (blue section). The blue area that is part of the alpha helix seen in the back, corresponds to Alanine 471.

BLOSUM 62 score means these aa substitutions were unlikely to occur. From the 41 aa positions listed in Table 3, only those with the five lowest ET ranks were selected: M68 (ET rank = 29.84), M212 (ET rank = 21.73), S236 (ET rank = 22.91), K298 (ET rank = 38.33) and N507 (ET rank = 29.53). Thus, the rationale behind our selection criteria for these final five aa (i.e., low ET rank plus a negative BLOSUM 62 score) was that, in spite of occupying positions predicted to be rather conserved and unlikely to differ between HtpB and GroEL (which are 75.5% identical), they still were different, suggesting a role in functional diversity. These five residues, however, were rather scattered (Figure 2C). M68 is in the equatorial domain, above the bottom chaperonin face involved in inter-ring contacts. M212, S236 and K298 are located in different parts of the apical domain: M212 is below the upper chaperonin face involved in substrate recognition, whereas S236 lies right adjacent to aa involved in substrate recognition on the chaperonin face opposite to where M212 is located, and K298 is near the intermediate domain, on the same face as S236. N507 is in the intermediate domain, just adjacent to some aa involved in intra-ring contacts. The scattered localization pattern of the 41 less-likely aa substitutions between HtpB and GroEL, did not allow us to pinpoint potential functional domains or structural clusters, with the exception of one cluster comprising positions 471 to 475 (equivalent to positions 470 to 474 in GroEL), which are situated on the outer face of the equatorial domain not involved in inter-ring contacts (Figure 2C). In spite of containing aa with intermediate/high ET ranks (between 109.10 and 185.86) this cluster was still of interest, mainly because it represents a surface domain predicted to form a protruding randomly coiled loop, sufficiently exposed to interact with other proteins (Figure 3). Therefore, 10 final aa positions were selected for functional testing through mutagenesis: the five scattered positions (M68, M212, S236, K298 and N507), plus the AEHKD cluster in positions 471-475.

### 3.2. Validation of the HtpB-hECM29 Yeast-Two-Hybrid Interaction as a Functional Reporter Assay

**Figure 4:**
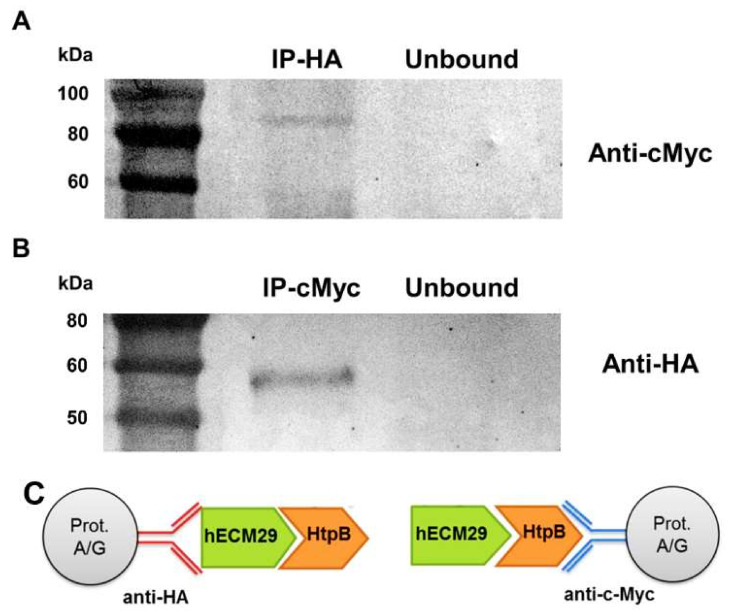
Confirmation of the physical interaction between HtpB and hECM29. (**A**) Immunoblot of the immunoprecipitation (IP) done with the HA-specific antibody (anti-HA). Total proteins from *S. cerevisiae* co-expressing c-Myc-HtpB and HA-hECM29 fusions were immuno-precipitated with anti-HA (IP-HA), separated by SDS-PAGE, transferred to a membrane, and immuno-stained with c-Myc-specific antibody (Anti-cMyc). An ~85-kDa band corresponding to the HtpB+Gal4 DNA binding domain+c-Myc tag fusion protein is seen in the immunoprecipitate (IP-HA), but not in the IP supernatant (Unbound). (**B**) Immunoblot of the IP done with anti-c-Myc, where total proteins from *S. cerevisiae* co-expressing c-Myc-HtpB and HA-hECM29 fusions were immuno-precipitated with anti-cMyc (IP-cMyc), and immuno-stained with anti-HA. An ~57-kDa band corresponding to the hECM29+Gal4 activating domain+SV40 nuclear-localization signal+HA tag fusion protein is seen in the immuno-precipitate (IP-cMyc), but not in the IP supernatant (Unbound). (**C**) Diagrams to aid in the interpretation of the immunoblots. The diagram on the left shows the HtpB-hECM29 complex captured with anti-HA immobilized on a protein A/G agarose bead (Prot. A/G); as in the assay corresponding to Panel A. The diagram on the right shows the HtpB-hECM29 complex captured with immobilized anti-c-Myc; as in the assay corresponding to Panel B. The mass of the protein standards shown at the left of panels A and B is given in kDa.

It was first confirmed through immunoprecipitation (IP) that HtpB and hECM29 indeed interact physically (Figure 4). IP also confirmed that hECM29 was correctly fused with the HA tag and the GAL4 activating domain. Then, it was also confirmed that in our yeast-two-hybrid (Y2H) plate assays only HtpB (but not GroEL) consistently interacts with hECM29 (Supplemental Figure S1). To confirm that the parental HtpB and GroEL baits to be used in Y2H assays were properly constructed and expressed, we first used restriction digestion and immunoblotting (Supplemental Figure S2). The sizes of the restricted fragments and the reactions with specific antibodies were as predicted, thereby confirming that wild-type (WT)-HtpB and WT-GroEL were expressed in the correct translational frame with the c-Myc and GAL4 DNA binding domain polypeptides (Figure S2).

Based on the aforementioned results of the ET analysis, we decided to make the following site-directed mutations on the 10 aa positions chosen for functional testing: (i) Single site-directed mutations performed in *htpB*, leading to alanine substitutions in 9 of the 10 chosen aa positions of HtpB (M68A, M212A, S236A, K298A, N507A, E472A, H473A, K474A and D475A). Since the HtpB aa in position 471 was already an alanine, it was not included. (ii) Multiple site-directed mutations done in *htpB* and *groEL*. HtpB mutant 472-475/A had alanine substitutions in four of the five HtpB aa clustered in positions 471-475 (again, A471 was not included). The proximity of the nucleotides to be changed allowed this multi-site mutation to be done using a single primer set (1415-17-18-20-21-24, forward and reverse). HtpB mutant MMSKN/A had simultaneous alanine substitutions in all 5 scattered aa positions and required one PCR reaction with five primers: M68A_R, M212A_R, S236A_R, K298A_R and N507A_R. HtpB mutant EGPGY had its five scattered aa positions (MMSKN), changed for those displayed in GroEL (EGPGY). This mutant also required five primers: M68E_R, M212G_R, S236P_R, K298G_R and N507Y_R. HtpB mutant KGGDG had its five clustered aa in positions 471-475 (AEHKD) changed for those displayed in GroEL (KGGDG). For this mutant, two primer sets were used in two separate steps. The first primer pair (1411-12-13-15_F/1411-12-13-15_R) changed the first two aa (A471K, E472G), and the second pair (1417-18-20-22-24_F/1417-18-20-22-24_R) changed the remaining three aa (H473G, K474D, D475G). Mutant GroEL-AEHKD had its five clustered aa in positions 470-474 (KGGDG) changed for those displayed by HtpB in positions 471-475 (AEHKD), and was generated using only one primer set: GroEL470-474_F/GroEL470-474_R. Mutant GroEL-MMSKN had its five scattered aa in positions 67, 211, 235, 297 and 506 (EGPGY) changed to those displayed by HtpB in positions 68, 212, 236, 298 and 507 (MMSKN), and was generated in one PCR reaction using five primers: E67M_R, G211M_R, P235S_R, G297K_R and Y506N_R. Finally, mutant GroEL-Multi involved all 10 selected aa positions (5 scattered + 5 clustered) and had its aa in positions 67, 211, 235, 297, 506 and 470-474, changed to the HtpB aa in equivalent positions. To generate the GroEL-Multi mutant, the pBluescript construct encoding the GroEL-MMSKN mutant was used as template to introduce the additional nucleotide changes corresponding to aa positions 470-474, using the single primer set GroEL470-474_F/GroEL470-474_R. DNA sequencing was used to confirm the correctness of the parental bait constructs, as well as the accuracy of the single and multiple site-directed mutations (Supplemental Figure S3). Some mutations led to changes in restriction sites, thereby allowing further confirmation through restriction digestions (Supplemental Figure S4).

### 3.3. Single- and Multi-Site Directed Mutations in Selected aa Positions Affect the Yeast-Two-Hybrid (Y2H) HtpB-hECM29 Interaction

**Figure 5:**
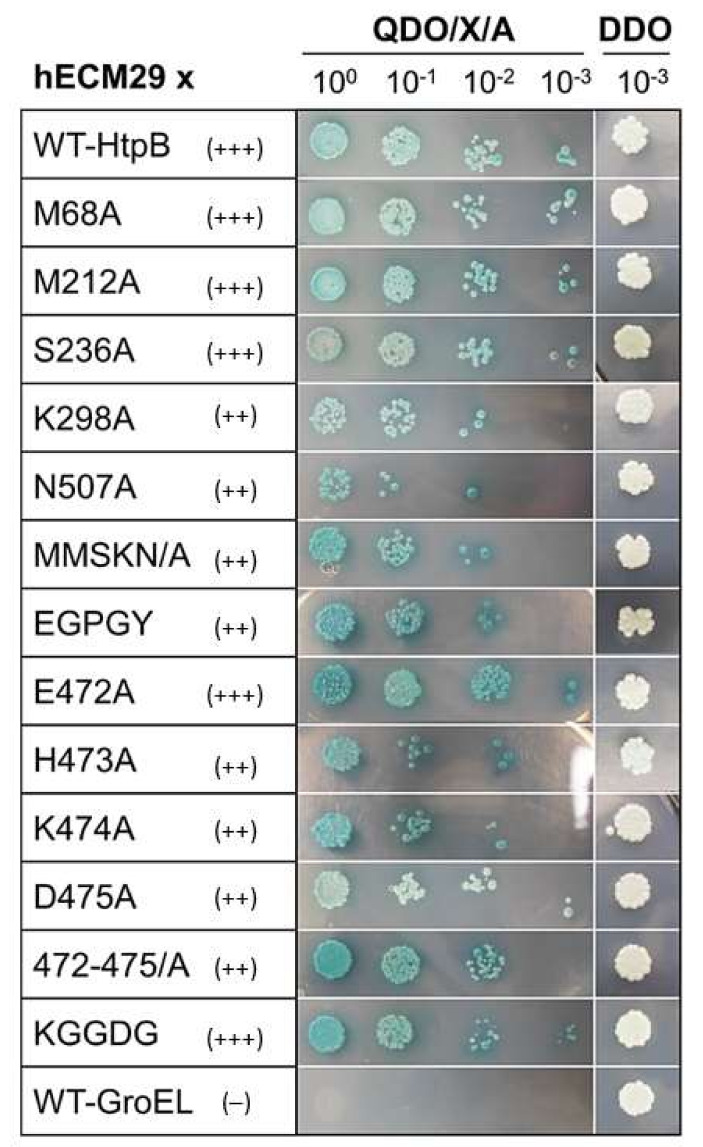
Effect of amino acid substitutions upon the HtpB-hECM29 interaction, evaluated in Y2H Plate assays. Images show the 5-day growth of yeast spotted on QDO/X/A plates, as well as the positive growth control spotted on DDO plates (far right column). The baits used to interact with hECM29 are listed in the first column. The positive interaction control is the wild-type HtpB bait (WT-HtpB), and the negative interaction control is the wild-type GroEL bait (WT-GroEL). Notice that for WT-GroEL, there is a shadow lawn in the 10^0^ dilution, but not in the 10^−1^ dilution. A shadow lawn is produced by yeast cells that, upon incubation, dry up on the agar plate surface, in the absence of defined colony growth. The interaction score for each bait is given as: (+++) = Positive, (++) = Impaired, and (–) = negative. The positive growth control was spotted with the highest dilution used (10^−3^), to ensure that any growth defect observed on QDO/X/A plates was not due to faulty inoculation. The images shown are representative of at least three independent experiments (all giving the same results).

In Y2H Plate Assays, the single-site HtpB mutants K298A and N507A (among the scattered aa positions), and H473A, K474A and D475A (among the clustered aa positions), showed impaired interactions with hECM29 (Figure 5). It is noteworthy that besides a growth reduction, mutant D475A also showed an obvious reduction in the color intensity of colonies. The multiple-site substitutions in the scattered HtpB positions 68, 212, 236, 298 and 507, for either alanine (HtpB mutant MMSKN/A) or the GroEL aa (HtpB mutant EGPGY), also showed impaired interactions (Figure 5). Of the two multiple-site mutations targeted to the HtpB aa cluster at positions 471-475 (HtpB mutants 472-475/A and KGGDG), only the former showed reduced growth, suggesting an impaired interaction of mutant 472-475/A with hECM29 (Figure 5). However, the colonies of both these cluster mutants appeared to be slightly bluer than those of the WT-HtpB control, suggesting an enhanced interaction. Similarly, the single mutation E472A appeared to enhance the HtpB-hECM29 interaction, judging from the more intense blue color of the colonies and the increased colony forming units (CFU) density (more evident in the 1:100 dilution spot), in relation to the WT HtpB bait control (Figure 5).

**Figure 6:**
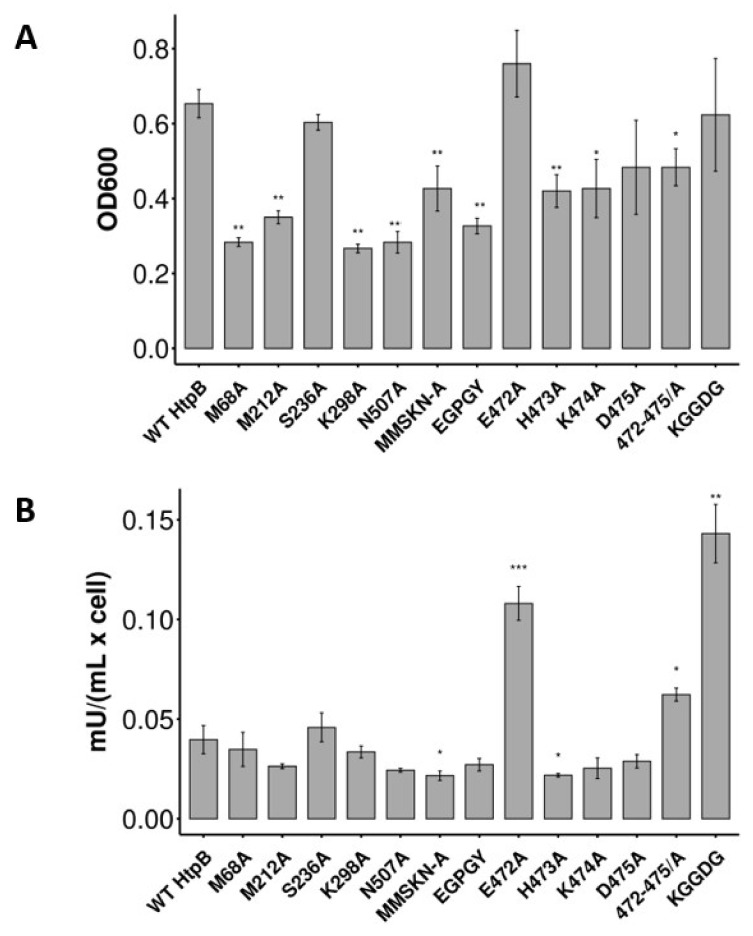
Bar graphs showing quantitative measurements of the HtpB-hECM29 interaction, evaluated in Y2H Broth assays. All quantitative measurements were taken after 6 days of incubation in QDO/A broth at 30 °C. Height of the bars represents the mean, and the error bars represent ± one standard deviation, from three independent measurements (*n* = 3). Asterisks indicate statistical significance in relation to the quantitative measurements of wild type HtpB (WT HtpB), with the following *p*-values: * <0.05, ** <0.01, *** <0.001. Statistical significance was calculated by the Student-*t* test. (**A**) Growth of yeast cells carrying the different HtpB baits indicated in the horizontal axis. Growth was measured as OD_600_ of the broth culture and is indicated in the Y axis. (**B**) Alpha-galactosidase activity of yeast cells carrying the different HtpB baits (indicated in the horizontal axis), is given in the Y axis as milli units per mL, per cell.

The quantitative optical density (OD_600_) measurements of Y2H Broth Assays (Figure 6A), confirmed all the results of the Y2H Plate Assays, including the growth enhancement (albeit not statistically significant) for mutant E472A, and the growth reduction of mutant 472-475/A. In addition, OD_600_ measurements also showed statistically significant growth impairments for the M68A and the M212A mutants (Figure 6A). The quantitative measurement of α-galactosidase activity (Figure 6B) clearly confirmed the impaired interaction of hECM29 with HtpB mutants MMSKN/A and H473A. Although reductions in α-galactosidase activity for HtpB mutants M68A, M212A, K298A, N507A, EGPGY, K474A and D475A correlated with the interaction impairments seen in Y2H Plate assays and(or) the quantitative OD_600_ measurements, such reductions were not statistically significant (Figure 6B). Importantly, the 472-475/A and KGGDG multi-site mutants showed statistically significant increases in α-galactosidase activity, confirming the observations from the Y2H Plate assays (i.e., a slight increase in colony color, Figure 5). It should be noted here that the reporter genes carried by the yeast strain Y2HGold respond to the transcription factor Gal4 through different promoters, implying that the levels of responsiveness of these reporters could be different (and independent) from each other, in particular when the stringency of the media used (e.g., quadruple drop-out [QDO] SD broth versus QDO/X/A agar) is considered. It is then possible that the interaction of hECM29 with HtpB multi-site mutants 472-475/A and KGGDG resulted in enhanced expression of α-galactosidase (which depends only on promoter M1), while growth (which requires the activation of promoters G1 and G2, needed for the biosynthesis of histidine and adenine, respectively) was either reduced or not affected. This could also explain, at least in part, why the expression of α-galactosidase by the E472A mutant bait was more enhanced (reaching statistical significance in the quantitative Y2H broth assays) than the rate of growth; or why the expression of α-galactosidase by the D475A mutant bait was seemingly more impaired than the rate of growth (Figure 5 and Figure 6).

**Figure 7:**
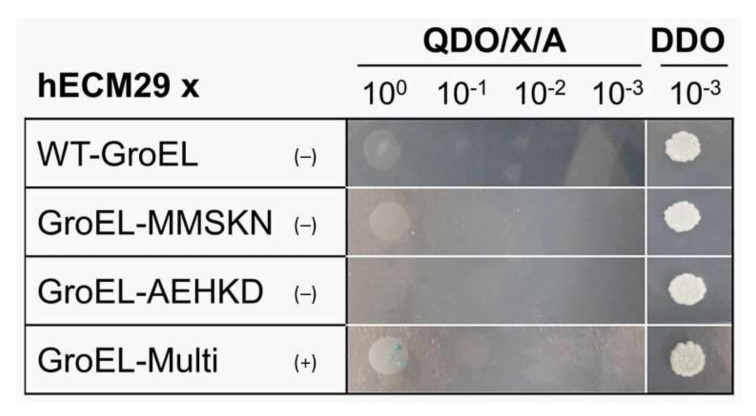
Effect of amino acid substitutions upon the GroEL-hECM29 interaction, evaluated in Y2H plate assays. Images show the 5-day growth of yeast spotted on QDO/X/A plates, as well as the growth of a positive control spotted on DDO plates (far right column). The baits used to interact with hECM29 are listed in the first column. The interaction score for each bait is given in parenthesis as follows: (+) = weak, and (–) = negative. The positive growth control was spotted with 10 μL of the highest dilution used (10^−3^), to ensure proper inoculation. The images shown are representative of at least three independent experiments (all giving the same results).

We also evaluated the effect of multi-site mutations in *groEL* through Y2H Plate Assays, albeit Broth Assays were not run for the GroEL mutant baits. As shown in Figure 7, the WT GroEL bait and both the GroEL MMSKN and GroEL AEHKD mutant baits, showed a negative interaction with hECM29. When all 10 selected aa positions were simultaneously changed in GroEL, for the HtpB aa in equivalent positions, mutant GroEL-Multi showed a weak (albeit consistent) interaction with hECM29, as judged from the unequivocal growth of distinct blue colonies in the undiluted spot, as well as the presence of a shadow lawn in the 10^–1^ spot (Figure 7). A shadow lawn is produced by yeast cells that, upon incubation, dry up on the agar plate surface, in the absence of colony growth. These results collectively suggest that both the scattered and the clustered aa positions chosen via the ET analysis, are involved (to different extents) in mediating the HtpB-hECM29 interaction.

In summary, residues K298, N507, H473 and K474 seemed to be functionally important, whereas M68, M212 and D475 played a minor role in the interaction with hECM29. A471 (and its equivalent GroEL residue K470) was indirectly tested, only as part of the multiple-site mutants HtpB-KGGDG and GroEL-AEHKD. S236 (individually) showed no experimental effect, and E472 seemed to have a functionally inverse role, upon the HtpB-hECM29 interaction. The fact that HtpB mutant baits only showed impaired interactions with hECM29 (rather than weak or negative), and that GroEL mutant baits only showed weak interactions (rather than positive), collectively indicate that the HtpB-hECM29 interaction likely involves other aa positions not tested here, although possibly identified in our ET analysis (e.g., Table 3).

## 4. Discussion

A fundamental concept in protein biochemistry is that the aa sequence of a given protein determines its structural and functional properties. Thus, our working hypothesis stemmed from this fundamental concept and stated that specific aa positions in HtpB must be linked to its particular functional traits. In fact, this fundamental concept is exemplified by two notorious reports. Firstly, the chaperonin of a symbiotic strain of the bacterium *Enterobacter aerogenes* was reported to act as an insect neurotoxin [56]. This toxic chaperonin (comprising 545 aa) differs from the non-toxic GroEL (comprising 548 aa) in only 11 aa positions, of which four (V100, N101, D338 and A471) are critical for toxicity. When the non-toxic GroEL was mutated to display the *E. aerogenes* aa at the four critical positions, it became toxic [56]. In the second case, the *Mycobacterium leprae* chaperonin only requires three aa in key positions (T375, K409, and S502) to form a threonine calalytic group responsible for protease activity [57].

Although it is still not possible to know all the functional traits of a protein based solely on aa sequence, it is possible to make functional predictions, or inferences, based on sequence comparisons with well-studied proteins of known function (experimentally determined through biochemical means). For this, a number of bioinformatics tools have been developed for the analysis of aa sequences, their differences, and their role in evolution, as well as for the study of structure–function relationships. One of these tools is a 3-step approach, primarily based on the Evolutionary Trace (ET) method, developed to predict the functional importance of aa positions, as exemplified by Madabushi et al., Rajagopalan et al. and Bonde et al. [58,59,60,61]. This approach helped us to identify aa positions in HtpB that could be linked to one (or more) of those unique functions of HtpB not shared by GroEL. Not being capable of considering the predicted importance of all the 137 aa that are different between HtpB and GroEL, and experimentally test them against the unique functions of HtpB, we chose to only focus on 10 selected aa positions, and test them against only one functional reporter test (the Y2H interaction of HtpB with hECM29), as a proof of concept for the usefulness of the 3-step ET approach in mapping functionally relevant HtpB aa positions.

One rationale behind the ET analysis is that the functional impact of aa that varies among divergent branches of evolution is greater than that of aa that vary among closely related proteins [37]. Thus, the more proteins from different lineages that can be included in the ET analysis, the more information that can be obtained about the evolutionary importance of each aa. Since 1373 Group I chaperonin sequences from many different bacterial lineages were included in the ET analysis, we are confident that the ET ranking of the HtpB aa reflects well their evolutionary and functional importance. Conserved aa positions of low ET rank were mostly found buried at the core of HtpB (data not shown). This is not surprising since conserved core aa keep the overall structure of the protein, while surface residues (exposed to different selective pressures) evolve more rapidly and are prone to mutate free of structural restrictions [33]. However, it was obvious from the surface representations of Figure 2A, that the majority of aa exposed on the outer HtpB surfaces still had intermediate/low ET ranks (i.e., they were rather conserved). Not surprisingly, many of the conserved aa clustered on HtpB’s outer areas related to protein folding (which is the essential function of chaperonins), particularly in those residues responsible for peptide recognition and ATP binding (Table 2 and Figure 2B). With some exceptions indicated in Table 2, the protein folding-related aa were well conserved between HtpB and GroEL, and thus, we had to look beyond this group of aa to pinpoint possible candidates that could be linked to HtpB’s protein folding-independent functions, e.g., interaction with eukaryotic cytoplasmic proteins like ECM29 [16]. The fact, thus, that the 10 aa positions selected through our ET analysis (perhaps with the exception of S236) were linked to the Y2H interaction between HtpB and hECM29 was satisfying and constituted sufficient proof-of-concept for the validity of the experimental approach used.

Single point mutations in residues K298, H473, K474 and N507 impaired HtpB-hECM29 interactions, whereas the E472A single-site and the KGGDG multi-site mutation appeared to enhance such interaction (Figure 5 and Figure 6). Interestingly, all these residues were located on the same side of HtpB (Figure 2C), strongly suggesting that this could be the face that interacts with hECM29. If this were the case, it would imply that HtpB could interact with hECM29 as either a monomer or a 7-mer, mainly because this face of HtpB would still be physically exposed after the intra-ring contacts have been established in the heptameric ring. As explained in the Results section, the HtpB-hECM29 interaction likely involves other aa positions not experimentally tested here. In this respect, there are several aa positions distributed around K298 and S236 (blue and purple areas on the exposed face of the apical domain in Figure 2C), corresponding to I295, Q300, K308, G312, E337, A340, E342, and A352 (Table 3), which constitute less likely aa substitutions between HtpB and GroEL. These positions are nicely distributed over the face that potentially interacts with hECM29, and could very well be some of those missing aa, but obviously, further mutational experiments would be needed to test this notion. Other additional mutational experiments derived from our results (to be tested in both Y2H Plate and Broth assays), could also be proposed as follows: (i) the direct testing of a single substitution of A471 in HtpB for a K residue (found in the equivalent GroEL position K470), (ii) testing of a single substitution of E472 for a G residue (found in the equivalent GroEL position G471), and (iii) instead of the GroEL-Multi mutant, create a GroEL mutant with multiple site aa substitutions in only those positions shown to be important for the HtpB-hECM interaction (i.e., K298, H473, K474 and N507).

E472 is equivalent to position A471 of the neurotoxic chaperonin of *Enterobacter aerogenes* mentioned above, which is one of the four aa required for toxicity [56]. It is thus remarkable that the E472A mutation (that substituted the glutamic acid present in HtpB for the very aa present in the toxic chaperonin) led to an enhanced interaction of HtpB with hECM29. The A/E substitution has a BLOSUM 62 score of 5, which indicates that it is likely to occur; thereby suggesting that under the right selective pressures, HtpB mutants displaying alanine in position 472 might not be difficult to naturally select. Mostly based on the quantitative results of the Y2H Broth assays, H473 seems to have a greater effect on the HtpB-hECM29 interaction than K298, K474 and N507. In addition, the effect of multi-site mutations involving the AEHKD cluster in positions 471-475 (i.e., the 472-475/A and KGGDG mutants), seemed to carry more weight in affecting the Y2H HtpB-hECM29 interaction than the multi-site mutations related to the five scattered positions (i.e., the MMSKN/A and EGPGY mutants). These observations correlate well with the organizational predictions for the AEHKD cluster, indicating its random coil structural flexibility, where H473 and K474 had the highest accessible surface area to accommodate molecular interactions (Figure 3). Furthermore, histidine is infrequently found in position 473 (or equivalent). That is, out of the 17 different residues that could be possibly found at that position in the 1373 chaperonins included in the MSA, histidine occupies the 11th place, which adds to the uniqueness of that aa position.

An additional level of complexity not experimentally addressed here is the possible role that post-translational modifications of HtpB could play in the interaction with hECM29. Unpublished results, using two-dimensional protein gel electrophoresis followed by protein identification by mass spectrometry [62], indicate that HtpB purified from *L. pneumophila* cells exists in many different forms of either the same mass (60-kDa) but different pI, or with both different mass and different pI. Obviously, these forms emerged through post-translational processing, suggesting that HtpB is prone to being cleaved and phosphorylated. At this point, we cannot rule out the fact that when expressed in yeast, HtpB could be cleaved, phosphorylated or glycosylated, and that these post-translational modifications could have played a role in the interaction with hECM29. In this case, then, the effect of some of the identified aa upon the HtpB-hECM29 interaction could have depended on how they would influence the occurrence of post-translational modifications, and not necessarily on their specific position in the aa sequence.

The very existence of multifunctional chaperonins poses two possible scenarios for their evolution: (i) ancestral multifunctional chaperonins evolved into specialized protein folders by gradually losing functions, implying that functional diversity is a residual evolutionary trait; or (ii) modern chaperonins evolved from an ancestral specialized protein folder by gradually acquiring new functions. In agreement with the aforementioned report by Yoshida et al. [56], our results with the GroEL-Multi mutant support the notion that HtpB evolved from a precursor chaperonin specialized in protein folding, through aa substitutions that led to functional gain. The selective pressures behind the emergence of these aa substitutions could be diverse. On the one hand, it could be argued that these HtpB substitutions evolved in the context of intracellular infections of amoeba (the natural hosts of *L. pneumophila*), where the emerged ability to interact with ECM29 could benefit the pathogen and result in a more successful infection. The C-terminus half of ECM29, which interacts with HtpB, is also the half that interacts with eukaryotic cellular motors and actin-related proteins [32], which in turn bind to actin microfilaments. Since HtpB interacts with soluble and polymerized actin [16], it could be speculated that HtpB and cellular motors could share some common actin-related domains, perhaps implicated in the binding to ECM29. In this respect, we could further speculate that the interaction of HtpB with ECM29 is directly or indirectly involved in the reorganization of cortical microfilaments of host cells, as well as the alteration of mitochondrial trafficking, two phenotypes affected by recombinant HtpB, or purified HtpB attached to polystyrene microbeads [18]. It is also known that *L. pneumophila* requires active proteasomes for optimal growth in some host cells [63]. This requirement seems to be related to the proteasome-mediated degradation of endosomal proteins (to be used as nutrients by *L. pneumophila*) [64,65] and(or) the degradation of those *L. pneumophila* virulence factors that must have a transient intracellular effect [66]. Since secreted HtpB can be found on the cytoplasmic side of the *Legionella*-containing vacuole (LCV) during intracellular infection [16], and since ECM29 seemingly couples 26S proteasomes to specific cellular compartments [32], it is possible for the HtpB-ECM29 interaction to contribute to the localization of the 26S proteasome to the LCV, enhancing protein degradation. In this view, the functional difference between HtpB and GroEL could be easily explained by the fact that *E. coli* is mostly a commensal bacterium and not an intracellular pathogen.

On the other hand, it is possible that the aa substitutions that led to the interaction of HtpB with ECM29 obey more to intrinsic selective pressures imposed by a particular *L. pneumophila*’s physiology, than to an adaptation to the intracellular environment where *L. pneumophila* replicates. Since chaperonins are part of the cellular machinery responsible for maintaining a healthy proteome in bacteria, it seems reasonable to speculate that chaperonins would interact with components of this machinery, including Hsp90, Hsp70, DnaJ, small heat shock proteins and proteases, as reviewed by Wickner et al. [67]. Interestingly, ECM29 seemingly interacts with the eukaryotic counterparts of chaperones DnaJ, Hsp70, Hsp90, and some small heat shock proteins [29,32,68], interactions that are also listed in the *Saccharomyces* Genome Database (https://www.yeastgenome.org (accessed on 24 December 2021)). Although there are not known homologs of ECM29 in bacteria, it is not unreasonable to speculate that some domains of ECM29 involved in interactions with proteasome components and molecular chaperones and are also involved in interactions with some chaperonins (including HtpB). In this respect, the reported genetic interaction between ECM29 and the Class II chaperonin CCT6 [69], as well as the physical interaction between ECM29 and the co-chaperonin Hsp10 (reported in the *Saccharomyces* Genome Database at https://www.yeastgenome.org (accessed on 24 December 2021)), are thus very relevant. In this view, the functional difference between HtpB and GroEL could then be explained by intrinsic physiological differences between *E. coli* and *L. pneumophila*, in relation to their molecular machineries used in proteome maintenance. Evidence of these physiological differences is the fact that HtpB and GroEL are not functionally exchangeable. That is, HtpB cannot complement temperature-sensitive *groEL* mutations in *E. coli* [53], and HtpB cannot be substituted for GroEL in *L. pneumophila* [54], further suggesting a divergent evolution.

In conclusion, the ET trace analysis performed here allowed us to identify specific aa that positively or negatively affected the HtpB-hECM29 interaction. Regardless of the evolutionary molecular mechanism leading to the ability of HtpB to interact with ECM29, or the significance of such interaction in the physiology and(or) pathogenesis of *L. pneumophila*, the results presented provide sufficient proof of the concept that functional differences between HtpB and GroEL can be mapped to specific aa positions.

## Figures and Tables

**Table 1:** Primers used. The primer’s name in alphabetical/numerical order, nucleotide sequence, and application are given. For those primers used in multi-site directed mutagenesis, their application is designated as “Mutagenesis-multi”. Nucleotides in boldface show the sequence of the restriction site given in parenthesis, and the boldface-underlined nucleotides are those mutated from the wild type/parental sequence, to achieve the desired amino acid substitutions.

Primer’s Name	5′ → 3′ Sequence	Application
BamHI-htpB_R	ATA**GGATCC**TTACATCATTCCGCCCATG (**BamHI**)	PCR and sequencing
D475A_F	GGTAGCTGAGCACAAAG**C**CAACTACGGTTTCAACG	Mutagenesis
D475A_R	CGTTGAAACCGTAGTTG**G**CTTTGTGCTCAGCTACC	Mutagenesis
EcoRI-htpB_F	CCG**GAATTC**ATGATAATGGCTAAAGAATTA CG (**EcoRI**)	PCR and sequencing
EcoRI-groEL_F	CGG**GAATTC**ATGGCAGCTAAAGACG (**EcoRI**)	PCR and sequencing
E67M_R	CACCATCTGCGCACCCATATT**CAT**GAACTTGTCTTCCAGTTCGAT	Mutagenesis-multi
E472A_F	TAGTAAACAAGGTAGCTG**C**GCACAAAGACAACTACGG	Mutagenesis
E472A_R	CCGTAGTTGTCTTTGTGC**G**CAGCTACCTTGTTTACTA	Mutagenesis
GroEL461_F	CCGACGAAACCGTAGGTAAA	Sequencing
GroEL470-474_F	CTGTTGTTGCTAACACCGTT**GC**AG**C**CG**C**CG**C**CGCCAACTACGGTTACAACGCAG	Mutagenesis-multi
GroEL470-474_R	CTGCGTTGTAACCGTAGTTGGCG**G**CG**G**CG**G**CT**GC**AACGGTGTTAGCAACAACAG	Mutagenesis-multi
GroEL1154_R	TAGCAGCACCCACTTTGATAA	Sequencing
G211M_R	TTCCAGTTCTACTGC**CAT**AGTTTCCGGCTTGTTGATGAAGTAAGG	Mutagenesis-multi
G297K_R	GATCTCTTCAGAGATCACGGT**TTT**GCCAGTCAGGGTTGCGATATC	Mutagenesis-multi
HtpB419_F	AAGACAGCAAAGCCATTG	Sequencing
HtpB1200_R	AGCATCTTCAACACGAGC	Sequencing
H473A_F	GTAGTAAACAAGGTAGCTGAG**GC**CAAAGACAACTACGGTTTCAA	Mutagenesis
H473A_R	TTGAAACCGTAGTTGTCTTTG**GC**CTCAGCTACCTTGTTTACTAC	Mutagenesis
K298A_F	AGCGATGTTGCAAGACATTGCTATTTTGACT**GC**GGGTCAAGTTATTTCT	Mutagenesis
K298A_R	AGAAATAACTTGACCC**GC**AGTCAAAATAGCAATGTCTTGCAACATCGCT	Mutagenesis
K298G_R	AGAAATAACTTGACCC**CC**AGTCAAAATAGCAATGTCTTGCAACATCGCT	Mutagenesis-multi
K474A_F	TAAACAAGGTAGCTGAGCAC**GC**AGACAACTACGGTTTCAACG	Mutagenesis
K474A_R	CGTTGAAACCGTAGTTGTCT**GC**GTGCTCAGCTACCTTGTTTA	Mutagenesis
MMBD_F	TCATCGGAAGAGAGTAGTAAC	Sequencing
MMBD_R	CCTAAGAGTCACTTTAAAATTTGTATAC	Sequencing
M68A_F	TGAGTTTGAGCATCGTTTC**GC**GAACATGGGCGCTCAAATG	Mutagenesis
M68A_R	CATTTGAGCGCCCATGTTC**GC**GAAACGATGCTCAAACTCA	Mutagenesis
M68E_R	CATTTGAGCGCCCATGTTC**TC**GAAACGATGCTCAAACTCA	Mutagenesis-multi
M212A_F	TTTATCAACAACCAGCAAAAC**GC**GAGCTGTGAACTTGAGCATCC	Mutagenesis
M212A_R	GGATGCTCAAGTTCACAGCTC**GC**GTTTTGCTGGTTGTTGATAAA	Mutagenesis
M212G	GGATGCTCAAGTTCACAGCTC**CC**GTTTTGCTGGTTGTTGATAAA	Mutagenesis-multi
N507A_F	CAAAGTAACCCGTATGGCTCTGCAA**GC**TGCAGCTTCTGTA	Mutagenesis
N507A_R	TACAGAAGCTGCA**GC**TTGCAGAGCCATACGGGTTACTTTG	Mutagenesis
N507Y_R	CTACAGAAGCTGCAT**A**TTGCAGAGCCATACGGG	Mutagenesis-multi
P235S_R	AGCTTCCAGAACCGACAGCATTTCGCGGAT	Mutagenesis-multi
SalI-groEL_R	AGTC**GTCGAC**TTACATCATGCCGCCCA (**SalI**)	PCR and sequencing
S236A_F	CAGTATTCGTGAAATGTTG**G**CCGTATTGGAAGGTGTTGC	Mutagenesis
S236A_R	GCAACACCTTCCAATACGG**C**CAACATTTCACGAATACTG	Mutagenesis
S236P	GCAACACCTTCCAATACGG**GC**AACATTTCACGAATACTG	Mutagenesis-multi
Y506N_R	CACAGAAGCTGCGT**T**CTGCAGAGCAGAACG	Mutagenesis-multi
1411-12-13-15_F	ATGAAGCTTCTGTTGTAGTAAACAAGGTA**AAA**G**G**GCACAAAGACAACTACGGTTTCAAC	Mutagenesis-multi
1411-12-13-15_R	GTTGAAACCGTAGTTGTCTTTGTGC**C**C**TTT**TACCTTGTTTACTACAACAGAAGCTTCAT	Mutagenesis-multi
1415-17-18-20-21-24_F	CTTCTGTTGTAGTAAACAAGGTAGCTG**C**G**GC**C**GC**AG**C**CAACTACGGTTTCAACGCTGCAACTGG	Mutagenesis-multi
1415-17-18-20-21-24_R	CCAGTTGCAGCGTTGAAACCGTAGTTG**G**CT**GC**G**GC**C**G**CAGCTACCTTGTTTACTACAACAGAAG	Mutagenesis multi
1417-18-20-22-24_F	CTGTTGTAGTAAACAAGGTAAAAGGG**GG**C**G**A**T**G**G**CAACTACGGTTTCAACGCTGCAACTG	Mutagenesis-multi
1417-18-20-22-24_R	CAGTTGCAGCGTTGAAACCGTAGTTG**C**C**A**T**C**G**CC**CCCTTTTACCTTGTTTACTACAACAG	Mutagenesis-multi

**Table 2:** HtpB amino acids likely involved in protein folding-related functions. Amino acids are given in single letter code, and the numeral that follows the letter indicates the position in the 550 amino acid sequence. The ET rank for each position is given in parentheses. For those positions in which the HtpB and GroEL amino acids are different, the GroEL amino acid is given in square brackets.

Protein Folding-Related Function			
Intra-Ring Contacts (Formation of Heptameric Rings)	ATP-Binding	Polypeptide Substrate Recognition	Inter-Ring Contacts (Formation of 14-mer Barrel)
L7(47.14) [V]	R14(6.33)	Y200(10.29)	D12(133.91)
A23(125.52) [V]	T31(1)	S202(6.66)	L15(113.12) [V]
R37(9.63)	M32(16.1) [L]	Y204(1.65)	K106(36)
N38(9.8)	G33(1)	F205(14.17)	A109(48.43)
V39(5.59)	P34(1)	R232(32.97)	A110(24.42)
V40(44.13)	K52(6.27)	L235(31.35)	G111(2.21)
L41(39.88)	D53(1)	L238(3.42)	M112(49.9)
E42(62.78) [D]	G54(1)	E239(9.22)	D435(146.41) [E]
K81(21.07)	D88(1)	A242(46.79)	R446(79.93)
D84(9.44)	T92(1.64)	L260(7.37)	R453(48.94)
N113(22.45)	I151(29.03)	T262(14.39)	E462(32.73)
M115(68.31)	S152(4.14)	V264(19.47)	S464(50.97)
N182(43.17) [T]	A153(23.45)	V265(32.63)	V465(42.35)
L184(66.68)	A384(8.56)	N266(1.49)	N468(101.41)
R198(10.2)	D399(1.72)	R269(10.8)	
N208(47.69) [K]	A407(3.87)	I271(54.8)	
E217(117.15)	G416(1)		
K227(3.43)	I455(8.15)		
R232(32.97)	N480(41.39)		
R246(79.05) [K]	A481(13.62)		
E253(14.91)	A482(88.43)		
E256(41.75)	I494(30.94)		
E258(12.87)	D496(1.4)		
K273(74.67)			
F282(7.64)			
D284(16.16)			
R285(3.19)			
R286(2)			
Y361(9.78)			
A385(35.43)			
E387(4.19)			
M390(22.23)			
A459(37.54) [C]			
T517(4.29)			
E519(25.31)			
C520(50.52)			
M521(64.93)			
V522(68.71)			
A523(69.18) [T]			

**Table 3:** Positions of the multiple sequence alignment (MSA), for which HtpB and GroEL amino acids (aa) are different and represent less likely substitutions predicted by a negative BLOSUM score. Of the 137 positions in which HtpB and GroEL aa differ, only the 41 with a negative BLOSUM 69 score are shown here. The five positions with low ET ranks (real value evolutionary trace rank, or rvET rank) are highlighted.

Aligment Position	HtpB (aa)	GroEL (aa)	Variability (N°) ^a^	Variability (aa) ^b^	rvET Rank	Blosum Score
3	M	A	10	A**M**VTSPGKLD	46.31	−1
19	A	R	15	RKE**A**SVQDNHTILMY	130.61	−1
65	H	D	10	DCN**H**ESQAKG	46.08	−1
68	M	E	13	ERKAQ**M**IPLNHSV	29.84	−2
105	H	L	14	LINA**H**MVCSFYTQR	66.77	−3
126	L	T	15	TEAKINVDQ**L**SGRHY	210.52	−1
137	K	V	13	V**K**IQSRHETNDLA	93.13	−2
161	A	K	15	KDNERQSLT**A**HGVXI	201.68	−1
209	Q	P	14	PSANTR**Q**KGVHLMD	120.55	−1
212	M	G	7	GQ**M**LARS	21.73	−3
214	C	V	7	VA**C**ITSG	76.14	−1
218	H	S	11	SDKENRQT**H**GA	115.53	−1
236	S	P	8	PH**S**TNGAQ	22.91	−1
295	I	T	7	T**I**VANCM	113.27	−1
298	K	G	10	GA**K**NDSQHRE	38.33	−2
300	Q	T	14	T**Q**EIVLSKHRMNDA	112.40	−1
308	K	M	17	MLRISFY**K**GANVTHCDQ	85.41	−1
312	G	K	12	KNTDSAQ**G**EHRM	116.02	−2
337	E	V	20	VALDNGMSKI.**E**FTRQHYCP	157.89	−2
340	A	E	17	ETSKP**A**QGDNVCRH.YL	169.34	−1
342	E	A	18	AQDSN**E**VKTRHMIGLPFY	187.52	−1
352	A	Q	14	Q**A**KSVTGNRHMILE	151.18	−1
424	Q	A	17	AYISTVLK**Q**REGMFHCN	149.47	−1
426	A	K	19	KPT**A**SVQDYEIGCLNRHMF	173.35	−1
428	D	A	18	ALESTKH**D**QVGFRN.ICP	191.20	−2
444	L	A	7	AVI**L**FMT	64.13	−1
445	R	L	16	LKIF**R**MEQVAYTSCGN	122.42	−2
457	T	L	18	LFHAVEIK**T**YNQSDRGCM	194.97	−1
461	Y	E	19	ELKF**Y**IDQAVGSMTHWRCN	142.12	−2
463	A	P	10	PG**A**SENDTRK	46.27	−1
469	K	T	14	T**K**RQANESHMIYDG	126.50	−1
471	A	K	14	KRLM**A**IQESVTGHY	115.70	−1
472	E	G	17	GNHSA**E**.KQTDRLMCVI	185.86	−2
473	H	G	17	G.RAKSNTEV**H**DQPLMC	109.10	−2
474	K	D	15	DPASE**K**Q.TVGNCRH	183.88	−1
475	D	G	20	GAVSLPKERYIT**D**QFNHW.M	184.40	−1
484	G	E	12	E**G**DNMKLFHRSA	82.24	−2
503	M	S	14	SCTIV**M**NAYLFQHG	65.83	−1
507	N	Y	11	Y**N**SHDKAFGLT	29.53	−2
530	E	A	15	AKGS.P**E**NDTVQIMH	219.66	−1
536	D	G	15	G.A**D**QSEMNPYHVTI	118.38	−1

^a^ Number of possible aa in the entire MSA that can be found at the corresponding position. ^b^ List of the “X” possible aa that can be found at the corresponding position, where the value of X is given in the Variability (No.) column. Amino acids are ordered by frequency of occurrence. The GroEL aa were the most frequently found, and the HtpB aa are shown in boldface to highlight their commonness or rarity. A period in the list means that a sequence gap is found in some chaperonins in the corresponding position.

## Supplementary Materials

The following are available online at https://www.mdpi.com/article/10.3390/biom12010059/s1, Figure S1: Valenzuela et al-FigS1-Confirmation by digestion-immunoblot, Figure S2: Valenzuela et al-FigS2-Confirmation of GroEL neg interact, Figure S3: Valenzuela et al-FigS3-Confirmation by DNA sequencing, Figure S4: Valenzuela et al-FigS4-Confirmation by Restriction digestions, Table S1: Valenzuela et al-TabS1-SD media, Table S2: Valenzuela et al-TabS2-Ranks 550aa.
