# Supplementary material for: The Functional Differences between the GroEL Chaperonin of Escherichia coli and the HtpB Chaperonin of Legionella pneumophila Can Be Mapped to Specific Amino Acid Residues
Source: Biomolecules. 2021 Dec 31;12(1):59. doi: 10.3390/biom12010059 (PMC8774168; doi:10.3390/biom12010059)
Supplement: Supplementary file 1 [file biomolecules-12-00059-s001.zip › Valenzuela et al-Suppl Figs in PP.pptx]

## Slide 1
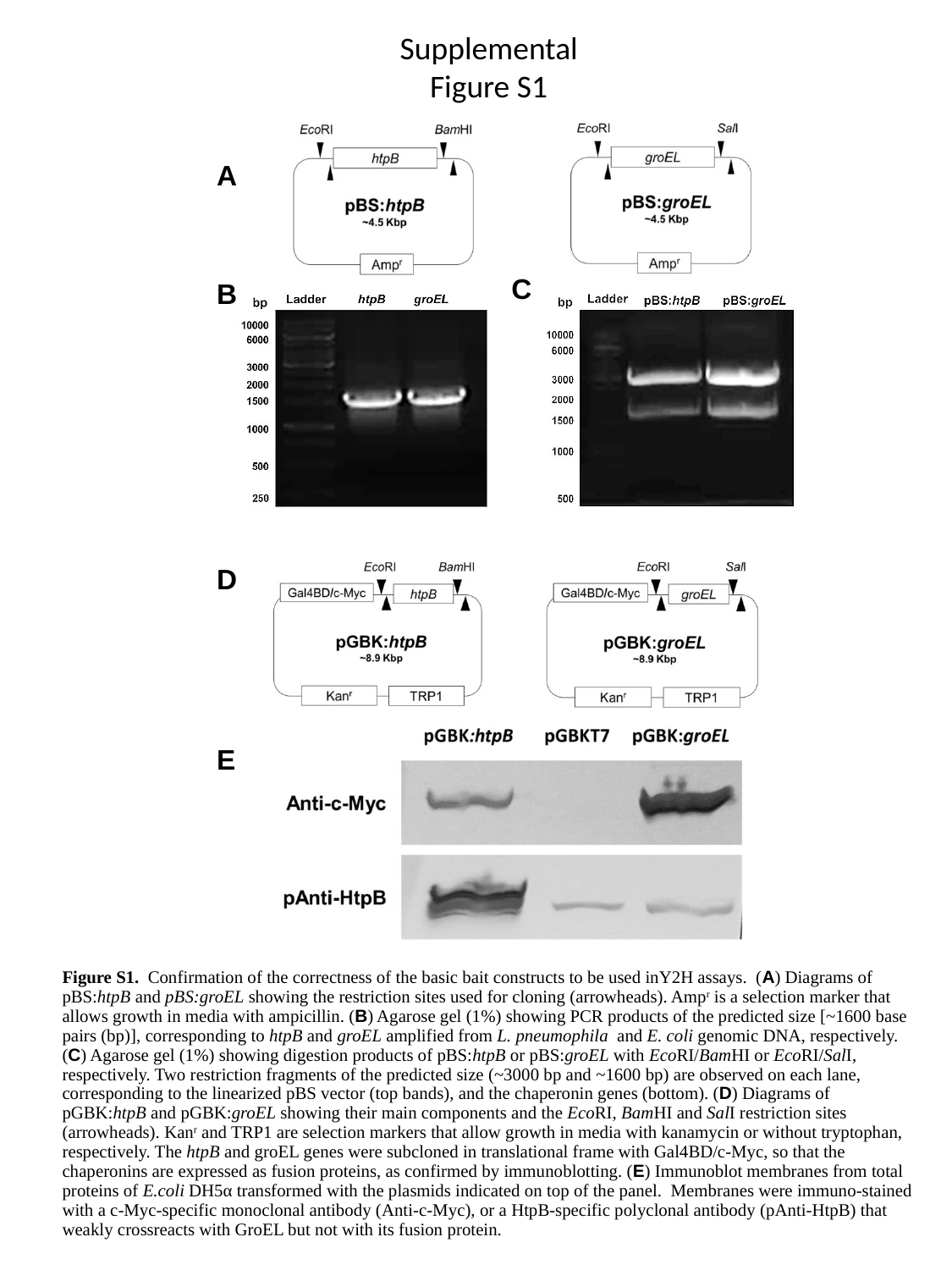

Supplemental
Figure S1
A
C
B
D
E
Figure S1. Confirmation of the correctness of the basic bait constructs to be used inY2H assays. (A) Diagrams of pBS:htpB and pBS:groEL showing the restriction sites used for cloning (arrowheads). Ampr is a selection marker that allows growth in media with ampicillin. (B) Agarose gel (1%) showing PCR products of the predicted size [~1600 base pairs (bp)], corresponding to htpB and groEL amplified from L. pneumophila and E. coli genomic DNA, respectively. (C) Agarose gel (1%) showing digestion products of pBS:htpB or pBS:groEL with EcoRI/BamHI or EcoRI/SalI, respectively. Two restriction fragments of the predicted size (~3000 bp and ~1600 bp) are observed on each lane, corresponding to the linearized pBS vector (top bands), and the chaperonin genes (bottom). (D) Diagrams of pGBK:htpB and pGBK:groEL showing their main components and the EcoRI, BamHI and SalI restriction sites (arrowheads). Kanr and TRP1 are selection markers that allow growth in media with kanamycin or without tryptophan, respectively. The htpB and groEL genes were subcloned in translational frame with Gal4BD/c-Myc, so that the chaperonins are expressed as fusion proteins, as confirmed by immunoblotting. (E) Immunoblot membranes from total proteins of E.coli DH5α transformed with the plasmids indicated on top of the panel. Membranes were immuno-stained with a c-Myc-specific monoclonal antibody (Anti-c-Myc), or a HtpB-specific polyclonal antibody (pAnti-HtpB) that weakly crossreacts with GroEL but not with its fusion protein.

## Slide 2
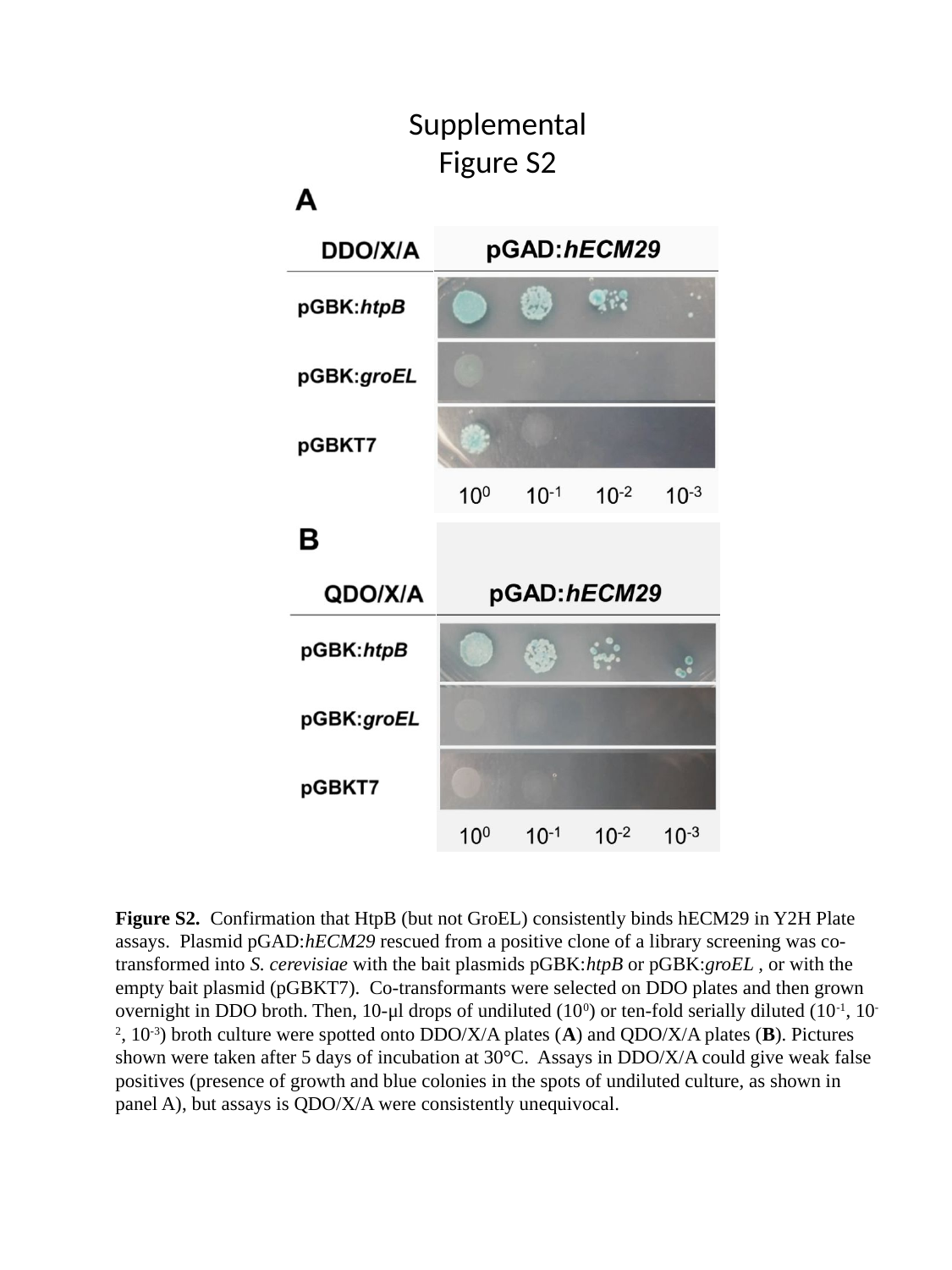

Supplemental
Figure S2
Figure S2. Confirmation that HtpB (but not GroEL) consistently binds hECM29 in Y2H Plate assays. Plasmid pGAD:hECM29 rescued from a positive clone of a library screening was co-transformed into S. cerevisiae with the bait plasmids pGBK:htpB or pGBK:groEL , or with the empty bait plasmid (pGBKT7). Co-transformants were selected on DDO plates and then grown overnight in DDO broth. Then, 10-μl drops of undiluted (100) or ten-fold serially diluted (10-1, 10-2, 10-3) broth culture were spotted onto DDO/X/A plates (A) and QDO/X/A plates (B). Pictures shown were taken after 5 days of incubation at 30°C. Assays in DDO/X/A could give weak false positives (presence of growth and blue colonies in the spots of undiluted culture, as shown in panel A), but assays is QDO/X/A were consistently unequivocal.

## Slide 3
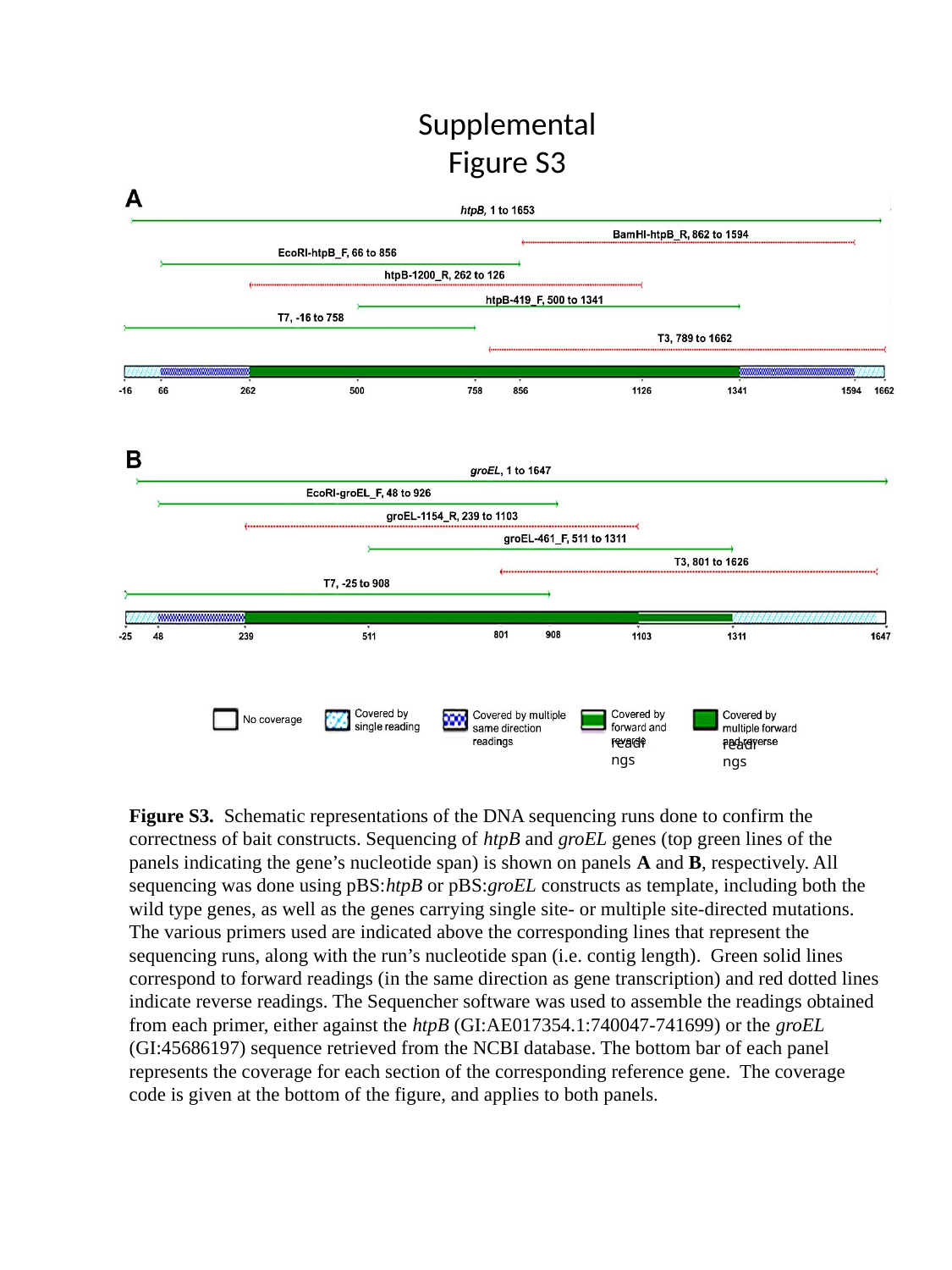

Supplemental
Figure S3
readings
readings
Figure S3. Schematic representations of the DNA sequencing runs done to confirm the correctness of bait constructs. Sequencing of htpB and groEL genes (top green lines of the panels indicating the gene’s nucleotide span) is shown on panels A and B, respectively. All sequencing was done using pBS:htpB or pBS:groEL constructs as template, including both the wild type genes, as well as the genes carrying single site- or multiple site-directed mutations. The various primers used are indicated above the corresponding lines that represent the sequencing runs, along with the run’s nucleotide span (i.e. contig length). Green solid lines correspond to forward readings (in the same direction as gene transcription) and red dotted lines indicate reverse readings. The Sequencher software was used to assemble the readings obtained from each primer, either against the htpB (GI:AE017354.1:740047-741699) or the groEL (GI:45686197) sequence retrieved from the NCBI database. The bottom bar of each panel represents the coverage for each section of the corresponding reference gene. The coverage code is given at the bottom of the figure, and applies to both panels.

## Slide 4
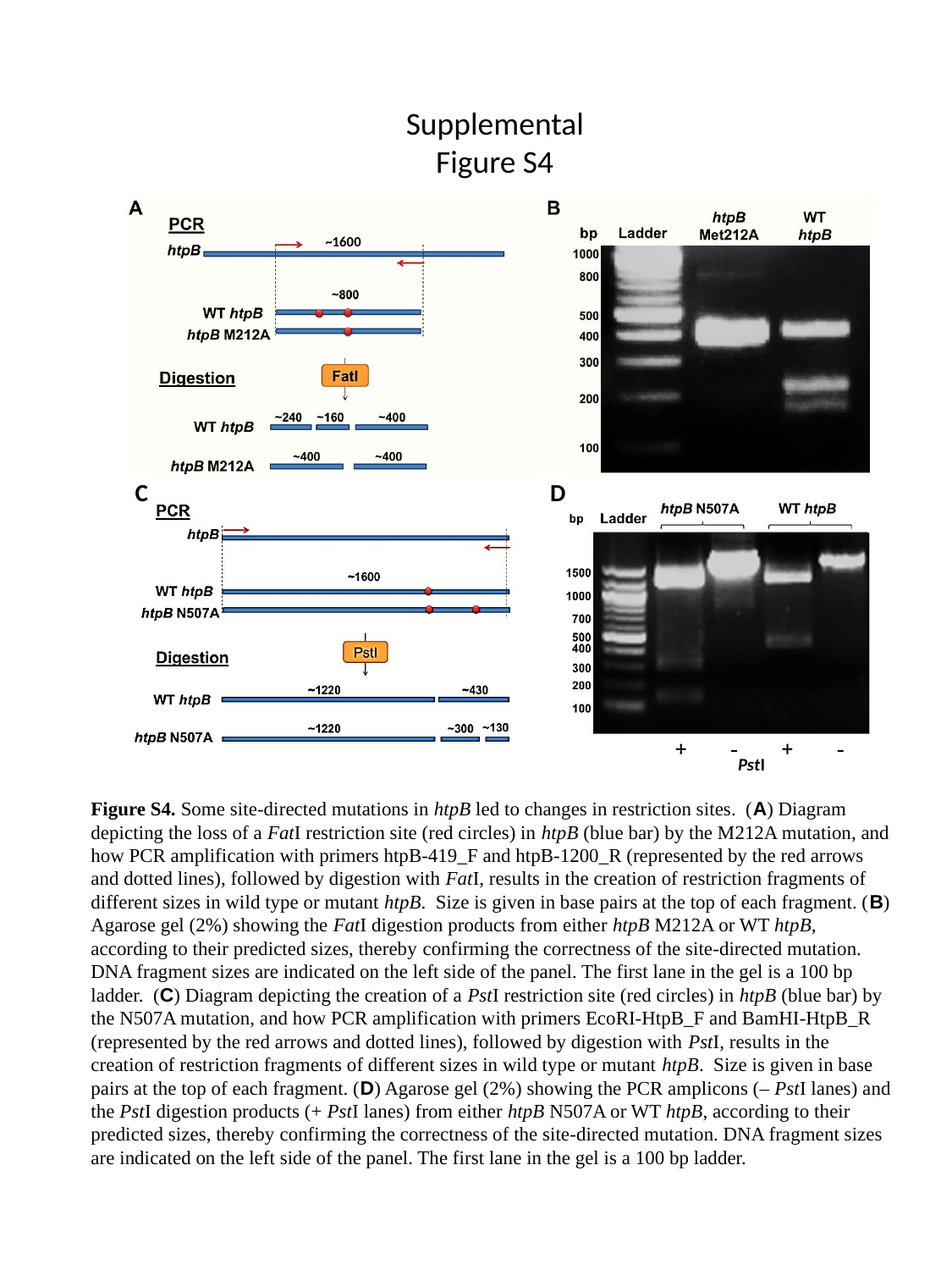

Supplemental
Figure S4
PstI
~1600
C
D
Figure S4. Some site-directed mutations in htpB led to changes in restriction sites. (A) Diagram depicting the loss of a FatI restriction site (red circles) in htpB (blue bar) by the M212A mutation, and how PCR amplification with primers htpB-419_F and htpB-1200_R (represented by the red arrows and dotted lines), followed by digestion with FatI, results in the creation of restriction fragments of different sizes in wild type or mutant htpB. Size is given in base pairs at the top of each fragment. (B) Agarose gel (2%) showing the FatI digestion products from either htpB M212A or WT htpB, according to their predicted sizes, thereby confirming the correctness of the site-directed mutation. DNA fragment sizes are indicated on the left side of the panel. The first lane in the gel is a 100 bp ladder. (C) Diagram depicting the creation of a PstI restriction site (red circles) in htpB (blue bar) by the N507A mutation, and how PCR amplification with primers EcoRI-HtpB_F and BamHI-HtpB_R (represented by the red arrows and dotted lines), followed by digestion with PstI, results in the creation of restriction fragments of different sizes in wild type or mutant htpB. Size is given in base pairs at the top of each fragment. (D) Agarose gel (2%) showing the PCR amplicons (– PstI lanes) and the PstI digestion products (+ PstI lanes) from either htpB N507A or WT htpB, according to their predicted sizes, thereby confirming the correctness of the site-directed mutation. DNA fragment sizes are indicated on the left side of the panel. The first lane in the gel is a 100 bp ladder.
