# Supplementary material for: The Functional Differences between the GroEL Chaperonin of Escherichia coli and the HtpB Chaperonin of Legionella pneumophila Can Be Mapped to Specific Amino Acid Residues
Source: Biomolecules. 2021 Dec 31;12(1):59. doi: 10.3390/biom12010059 (PMC8774168; doi:10.3390/biom12010059)
Supplement: Supplementary file 1 [file biomolecules-12-00059-s001.zip › Valenzuela et al-Suppl Table S1.pdf]

Supplementary Table S1. Preparation of Synthetic Defined (SD) media[a].

Component	Sigma Cat. No.	Amount (per L)	Type of SD medium					
			SD/-Leu	SD/-Trp	DDO	QDO	QDO/A	QDO/X/A
Yeast nitrogen base without aa	Y-0626	7 g	+	+	+	+	+	+
Glucose	G-8270	20 g	+	+	+	+	+	+
Adenine hemisulfate salt	A-3159	20 mg	+	+	+	–	–	–
Uracil	U-0750	12 mg	+	+	+	+	+	+
L-Arginine HCl	A-5131	20 mg	+	+	+	+	+	+
L-Histidine HCl monohydrate	H-8125	20 mg	+	+	+	–	–	–
L-Isoleucine	I-2752	20 mg	+	+	+	+	+	+
L-Leucine	L-8000	20 mg	–	+	–	–	–	–
L-Lysine HCl	L-5626	20 mg	+	+	+	+	+	+
L-Methionine	M-9625	20 mg	+	+	+	+	+	+
L-Phenylalanine	P-2126	30 mg	+	+	+	+	+	+
L-Threonine	T-8625	20 mg	+	+	+	+	+	+
L-Serine	S-4500	20 mg	+	+	+	+	+	+
L-Tryptophan	T-0254	30 mg	+	–	–	–	–	–
L-Tyrosine	T-3754	20 mg	+	+	+	+	+	+
L-Valine	V-0500	90 mg	+	+	+	+	+	+
Aureobasidin A[b,c]	630466[b]	200 µg	–	–	–	–	+	+
X-α-Gal[b,c]	630463[b]	40 mg	–	–	–	–	–	+

[a] These media can be prepared liquid, or solidified with 15 g/L bacteriological agar. QDO/X/A was only prepared in solidified form.

[b] These media components were not from Sigma-Aldrich, and the Catalog Nos. given are from Clontech.

[c] Aureobasidin A stock solution had 1 mg dissolved in 2 ml of absolute ethanol (500 µg/ml) and was stored at 4°C. X-α-Gal stock solution had 100 mg dissolved in 5 mL of dimethylformamide (20 mg/mL) and was stored at –20°C in the dark.
